# Rapid and Highly Stable Membrane Reconstitution by LAiR Enables the Study of Physiological Integral Membrane Protein Functions

**DOI:** 10.1021/acscentsci.2c01170

**Published:** 2023-02-22

**Authors:** Albert Godoy-Hernandez, Amer H. Asseri, Aiden J. Purugganan, Chimari Jiko, Carol de Ram, Holger Lill, Martin Pabst, Kaoru Mitsuoka, Christoph Gerle, Dirk Bald, Duncan G. G. McMillan

**Affiliations:** † Department of Biotechnology, Delft University of Technology, 2628 CD Delft, The Netherlands; ‡ Biochemistry Department, Faculty of Science, King Abdulaziz University, Jeddah 21589, Saudi Arabia; § Amsterdam Institute for Life and Environment (A-LIFE), AIMMS, Vrije Universiteit Amsterdam, 1081 HV Amsterdam, The Netherlands; ∥ Institute for Integrated Radiation and Nuclear Science, Kyoto University, Kyoto, 606-8501, Japan; ¶ Research Center for Ultra-High Voltage Electron Microscopy, Osaka University, Ibaraki, Osaka 565-0871, Japan; # Institute for Protein Research, Osaka University, Suita, Osaka 565-0871, Japan; □ Life Science Research Infrastructure Group, RIKEN SPring-8 Center, Kouto, Hyogo 679-5148, Japan; ● Department of Applied Chemistry, Graduate School of Engineering, The University of Tokyo, Bunkyo City, Tokyo 113-8654, Japan

## Abstract

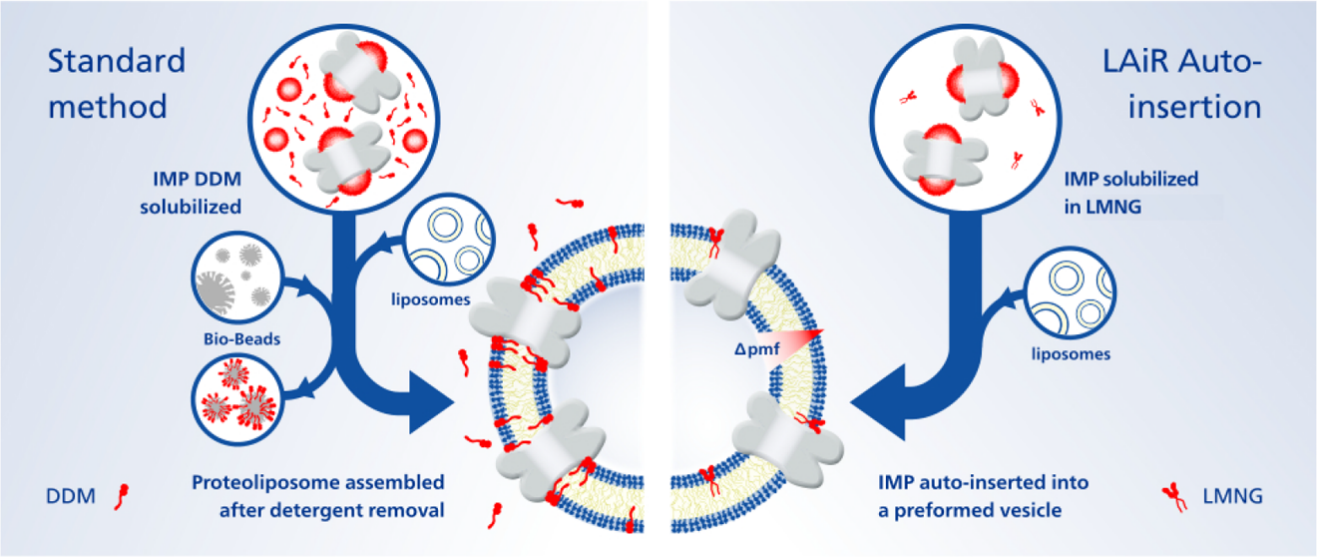

Functional reintegration into lipid environments represents a major challenge for *in vitro* investigation of integral membrane proteins (IMPs). Here, we report a new approach, termed LMNG Auto-insertion Reintegration (LAiR), for reintegration of IMPs into lipid bilayers within minutes. The resulting proteoliposomes displayed an unprecedented capability to maintain proton gradients and long-term stability. LAiR allowed for monitoring catalysis of a membrane-bound, physiologically relevant polyisoprenoid quinone substrate by *Escherichia coli* cytochromes *bo*_3_ (c*bo*_3_) and *bd* (c*bd*) under control of the proton motive force. LAiR also facilitated bulk-phase detection and physiological assessment of the “proton leak” in c*bo*_3_, a controversial catalytic state that previously was only approachable at the single-molecule level. LAiR maintained the multisubunit integrity and higher-order oligomeric states of the delicate mammalian F-ATP synthase. Given that LAiR can be applied to both liposomes and planar membrane bilayers and is compatible with IMPs and lipids from prokaryotic and eukaryotic sources, we anticipate LAiR to be applied broadly across basic research, pharmaceutical applications, and biotechnology.

## Abstract 2

LAiR: A low concentration LMNG based method for membrane auto-insertion, enabling rapid and stable lipid bilayer reconstitution of integral membrane proteins into liposomes and planar bilayers.

## Introduction

Membrane-embedded proteins fulfill a broad range of essential tasks in all living organisms, including cellular signaling and recognition, energy conversion, and transport of ions and metabolites. In line with these key biological functions, integral membrane proteins (IMPs) are highly represented among the targets of clinically utilized pharmacophores.^1,2^ Yet, even after decades of efforts, multisubunit IMPs still remain extremely challenging to study.

Functional investigation of IMPs typically requires purification of the protein from a complex mixture, thereby avoiding interfering signals from other membrane components. For IMP purification, detergents are commonly utilized and are viewed as simply a means of extracting the proteins from biological membranes and to retain the IMP solubilized in an aqueous buffer solution. To maintain a membrane mimicking environment, which may be needed for optimal function of a solubilized IMP,^3^ amphiphilic polymers (amphipols) or lipid bilayers encircled by amphiphilic protein scaffolds (nanodiscs) can be utilized.^4,5^ However, vectorial IMP functions, such as transmembrane signaling, maintenance of ion gradients, and transport of ions or metabolites, cannot be assessed in the detergent-solubilized or nanodisc-solubilized state.

Reintegration of purified membrane proteins into liposomes is a powerful tool to reconstitute and assess vectorial IMP properties and therefore widely utilized for functional studies.^6^ To facilitate incorporation of detergent-solubilized IMPs into the liposome, various approaches have been applied, including dialysis, gel filtration chromatography, rapid dilution, and adsorption of the detergent on the surface of polystyrene beads^7,8^ or combinations of these approaches.^9^ Bio-beads SM-2 resin (Bio-Rad, referred to as “Bio-beads” hereafter), a nonpolar polystyrene adsorbent, have been applied for liposome reintegration of a broad variety of IMPs, including bacterial transport proteins,^10^ ATPases from prokaryotic and eukaryotic sources,^11,12^ plant photosystems,^13,14^ and viral membrane proteins.^15^ This method is exceptionally efficient for removal of detergents with a low critical micelle concentration, such as Triton X-100 and β-dodecyl-maltoside (DDM).^8^ DDM presently is the most widely used detergent for purification and crystallization of IMPs^16^ and liposome reintegration of DDM-solubilized IMPs by Bio-beads may be regarded as a “gold standard” in the field. However, even in successful cases the resulting proteoliposomes suffer from ion leakiness and instability, preventing accurate measurement of vectorial functions and inability to accurately evaluate potential inhibitors.

In this report, we present a new approach for efficient and stable reintegration of IMPs into preformed unilamellar liposomes without the need to destabilize the liposomes by use of detergents that require removal during IMP reintegration. This approach, termed **L**MNG **A**uto-**i**nsertion **R**eintegration (LAiR), allows for proteoliposome formation at an unprecedented rate, reintegration of IMPs is essentially completed within minutes.

LAiR reconstituted the impact of the physiological proton motive force on the activity of both cytochrome *bo*_3_ (c*bo*_3_) and cytochrome *bd* (c*bd*) from *E. coli* with a membrane-bound quinone as substrate. LAiR also enabled investigation of the proton “leak state” in c*bo*_3_, a state that is regarded as controversial, likely due to insufficient performance of standard IMP reintegration systems. LAiR succeeded in preserving long-term functional integrity and the higher-order oligomeric state of the fragile mammalian F-ATP synthase. The enzymatic activity of all tested membrane proteins and the structural stability of the proteoliposomes clearly exceeded the standard Bio-beads approach over extended periods. Finally, LAiR is highly compatible with both preformed liposomes and state-of-the-art membrane bilayers tethered on an electrode surface. Our method is highly reproducible and functions in all downstream applications tested to date. For these reasons, we expect LAiR to be broadly utilized as a new platform for efficient investigation of IMPs in a lipid environment.

## Results

### LMNG Auto-insertion Reintegration (LAiR) is Rapid and Provides Structurally Stable Proteoliposomes Capable of Maintaining Proton Gradients for Extended Time Periods

Two distinct methods have seen popularity for the removal of detergents to facilitate the reintegration of detergent-solubilized IMPs into liposomes. Either Bio-beads are added successively or a rapid dilution (typically 200-fold) is used.^7,8^ Other than the detergent the protein is isolated with, both methods also use the addition of the detergent *n*-octyl-β-d-glucopyranoside to the liposome preparation at elevated concentrations (typically ∼50 mM) to aid in IMP reintegration. In a second step these standard methods use either a large dilution step or polystyrene beads to effectively leach detergent from the reintegration reaction and minimize detergent in the resulting proteoliposome preparation. However, although detergents are essential in extracting an IMP from a biological membrane, they also have destructive properties. The most sensitive proteins to detergent harm are multisubunit IMPs in which lipid plays an important stabilizing role.^3^ Even more sensitive and difficult to handle are IMPs that interact with hydrophobic substrates such as quinones.^17,18^

Lauryl maltose neopentyl glycol (LMNG) has been described as suitable for preserving IMP stability and activity^19^ and for preparing IMPs for structural analysis by X-ray crystallography^19^ and cryo electron microscopy (cryo-EM).^20^ LMNG’s lipid like molecular architecture conveys it with an extraordinarily low critical micelle concentration^19^ and high affinity for IMPs in solution.^21^ These properties allow as little as 0.002% LMNG to retain an IMP in solution, as compared to ∼0.025% needed in case of DDM, the currently most widely used detergent.^16^ We reasoned that usage of LMNG would reduce the impact of residual detergent on the integrity of reconstituted proteoliposomes.

**Figure 1:**
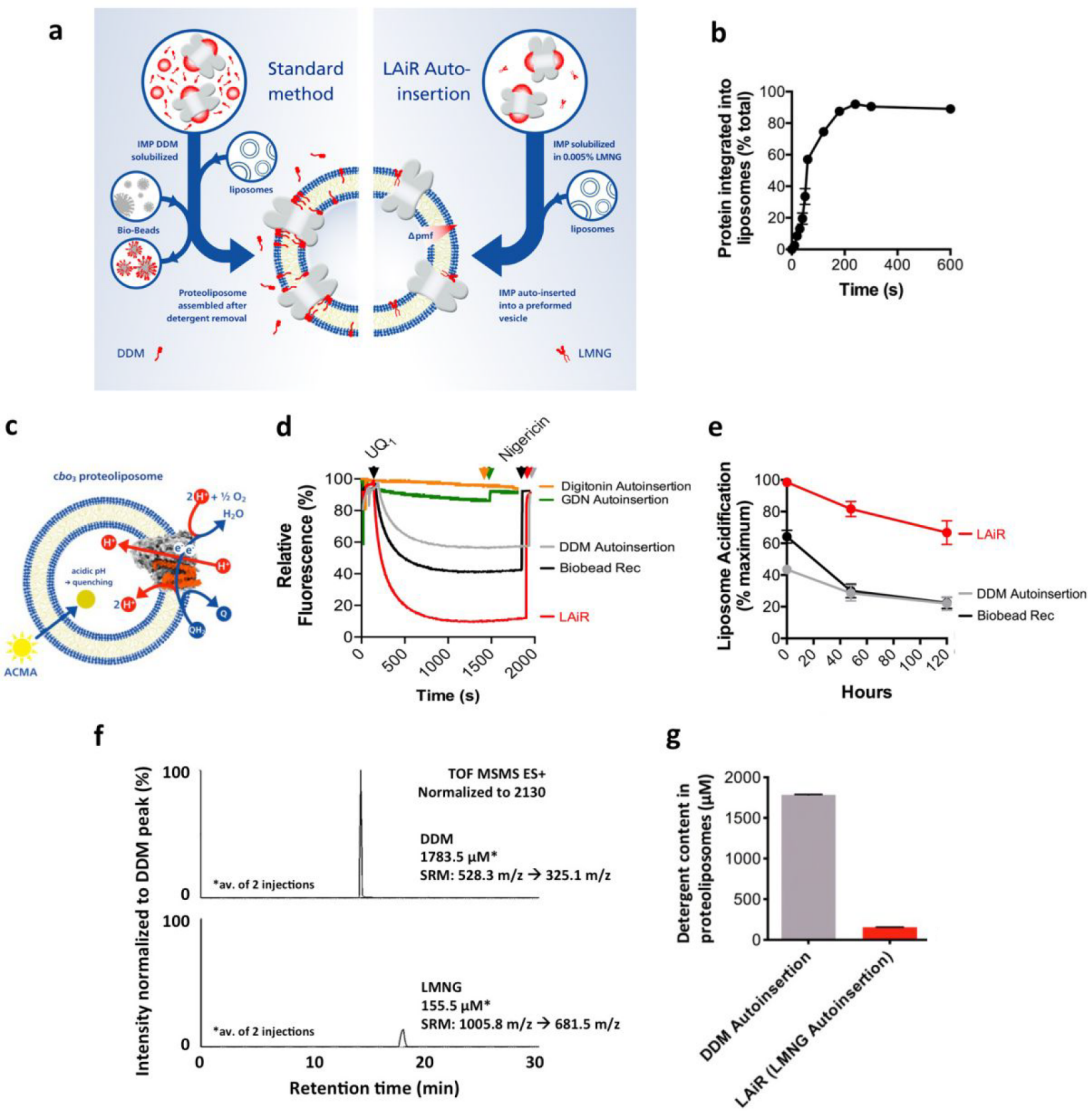
|LMNG Auto-insertion Reintegration (LAiR) results in highly stable proteoliposomes compatible with strong proton gradients. a, Schematic view of the standard reintegration method, where Bio-beads are used to reduce detergent concentration, in comparison with the LAiR method, where the ultralow CMC detergent LMNG is used. b, Time course of reintegrating c*bo*_3_ purified using LMNG into *E. coli* polar lipids liposomes using auto-insertion. c, Scheme of the experimental system used in panel d to assess proton pumping activity by reintegrated c*bo*_3_. The reaction was started by addition of soluble ubiquinone-1 (UQ_1_) that is reduced by addition of dithiothreitol. As protons are pumped into the proteoliposome lumen, the membrane-permeable fluorescent dye ACMA is accumulated inside the vesicles, which causes quenching of its fluorescence. This gradient was collapsed using nigericin to release the protons from the proteoliposome lumen, restoring full ACMA fluorescence. d, Liposome acidification by reintegrated c*bo*_3_ monitored by quenching of the ACMA fluorescence. Ubiquinol-1 (UQ_1_H_2_) was used to start the reaction. c*bo*_3_ proteoliposomes were prepared using either LAiR (red curve), Bio-beads/DDM (black curve), DDM auto-insertion (gray curve), GDN auto-insertion (green curve), or digitonin auto-insertion (orange curve). For the auto-insertion experiments, liposomes were incubated with the c*bo*_3_ sample for 30 min e, Cytochrome *bo*_3_ proteoliposome lifetime. Proteoliposomes were produced by either LAiR (red line, circles) or DDM auto-insertion (gray line, circles), by the Bio-beads/DDM method (black line, circles), or by rapid dilution reconstitution (gray line, squares). The proteoliposomes were stored on ice at 4 °C over a period of 120 h (5 days). ACMA quenching assays were performed on days 1, 3, and 5 to assess proteoliposome quality versus time. The maximum degree of quenching was calculated and presented relative to the LMNG auto-insertion sample on day 0. f and g, Quantification of detergent remaining after completion of either DDM auto-insertion or LAiR. f, Residual detergent in proteoliposomes was quantified using LC/MS (selected reaction monitoring). The upper panel shows residual detergent from DDM auto-insertion and the lower panel shows residual detergent from LAiR. g, is a graphical comparison. For each experiment 3 biological replicates (b, e), 2 technical replicates (f, g) or representative results (d, f) are shown with average values with error as a standard deviation of the mean.

Initially, we examined if an LMNG-purified IMP can insert into liposomes without the addition of *n*-octyl-β-d-glucopyranoside and subsequent detergent removal. We tested this approach using an LMNG preparation of the quinone-binding IMP cytochrome *bo*_3_ (c*bo*_3_ in solution containing 0.005% LMNG) from *Escherichia coli* and unilamellar liposomes composed of the cognate *E. coli* polar lipids. We coin this approach LMNG Auto-insertion Reintegration (LAiR) (Figure 1a; see “LAiR” in Methods for specific conditions). Intriguingly, we found that c*bo*_3_ almost completely inserted into the liposomes within <5 min (Figure 1b). In contrast, the total time needed for reintegration with Bio-beads or using the rapid dilution method exceeded 1 h and the efficiency of reintegration was substantially lower (Figure S1).

We then assessed the ability of the employed reintegration method to allow formation of proton gradients across the membranes of the proteoliposome. c*bo*_3_ is an enzyme that transfers electrons from a quinol-type substrate onto molecular oxygen resulting in the evolution of water. This membrane-based electron transfer activity is coupled to trans-membrane proton translocation^18^ (Figure 1c). We monitored the acidification of the proteoliposome lumen using a pH-sensitive fluorophore and soluble nonpoly isoprenoid ubiquinol-1 (UQ_1_) as an electron donor (Figure 1c). c*bo*_3_ proteoliposomes prepared using LAiR were capable of maintaining a 40% higher level of acidification as compared to the DDM purified, Bio-beads inserted standard (Figure 1d). Proteoliposome internal acidification caused by translocation of protons into the lumen was completely reverted by addition of the H^+^/K^+^ antiporter nigericin (Figure 1d), in line with a proton-tight proteoliposome system. Notably, c*bo*_3_ proteoliposomes prepared using LAiR displayed high stability over extended times. After storage on ice for 5 days, LAiR prepared c*bo*_3_ proteoliposomes still achieved ∼70% of the initially achieved maximum acidification of the liposomal lumen (Figure 1e). In contrast, five-day incubation of c*bo*_3_, reintegrated by either the standard Bio-beads method or rapid-dilution resulted in only ∼20% of the maximum activity measured for c*bo*_3_ proteoliposomes prepared using LAiR (Figure 1e).

These results show that LAiR is an extremely rapid technique for the reintegration of IMPs into stable proteoliposomes that is superior to current standard methods and compatible with long-term investigation.

### The Impact of Detergent Type on Reintegration of IMPs by “Auto-insertion”

In order to gain insight into molecular factors that determine reintegration efficiency and performance of the reintegrated IMP in auto-insertion into liposomes that are not predestabilized, we interrogated the role of the employed detergent type. We first assessed auto-insertion with DDM. A DDM-purified c*bo*_3_ sample (c*bo*_3_ in solution containing 0.05% DDM) inserted into preformed unilamellar liposomes with similar efficiency as observed with LAiR (Figure S2; also see “Auto-insertion” in Methods for specific conditions); however, the resulting proteoliposomes maintained only a relatively low level of luminal acidification (Figure 1d). We then investigated if the high level of acidification observed using LAiR correlated with the amount of residual detergent. We found an over 10-fold lower amount of residual detergent for c*bo*_3_ proteoliposomes prepared using LAiR as compared to the proteoliposomes prepared using DDM auto-insertion (Figure 1f,g). The 1 to 10 ratio of residual detergent in the resulting proteoliposomes is identical with that of detergent present in the c*bo*_3_ preparation, suggesting that all detergent present in the mixture inserts into the liposomes. Next, we assessed auto-insertion of c*bo*_3_ solubilized in digitonin, a detergent frequently used for very fragile IMPs such as supercomplexes.^22^ Contaminants in digitonin-purified *cbo*_3_ precluded quantitative assessment and comparison, so buffer exchange of LMNG-purified *cbo*_3_ was used. Strikingly, auto-insertion of digitonin-solubilized c*bo*_3_ (c*bo*_3_ in solution containing 0.02% digitonin; see “Auto-insertion” in Methods for specific conditions) yielded proteoliposomes that did not display significant liposome acidification (Figure 1d).

The degree of liposome acidification observed for LMNG, DDM, and digitonin increases with decreasing critical micelle concentration of the respective detergent (0.01 mM for LMNG,^19^ 0.17 mM for DDM,^23^ and 0.25–0.5 mM for digitonin^23^). We therefore decided to test if the critical micelle concentration is the determining factor for the observed varying levels of acidification in the produced proteoliposomes. For this aim we evaluated auto-insertion with glycol-diosgenin (GDN), a detergent that recently has seen popularity in particular for cryo electron microscopy applications.^24^ GDN harbors the same polar dimaltose hydrophilic headgroup as LMNG but comprises a steroid moiety as the hydrophobic tail instead of the two *n*-decyl hydrocarbon chains present in LMNG (Figure S3). In terms of critical micelle concentration (0.018 mM^25^), GDN resembles LMNG. Autoinsertion of c*bo*_3_ purified with GDN (c*bo*_3_ in solution containing 0.005% GDN; see “Auto-insertion” in Methods for specific conditions) resulted in proteoliposomes that diplayed only marginal lumen acidification (Figure 1d), revealing that the observed proteoliposome luminal acidification is not merely a function of the critical micelle concentration of the detergent used for auto-insertion.

Collectively these results demonstrate that LMNG performs clearly superiorly to other detergents with a low critical micelle concentration known for their mildness in solubilizing fragile IMPs. The specific combination of a dimaltose polar headgroup with a tail comprised of two linear hydrocarbon chains in LMNG apparently constitutes an important factor for achieving the apparent high stability of c*bo*_3_ proteoliposomes, as evidenced by the level of proteoliposomal lumen acidification (Figure 1d).

### LAiR Retains High Activity with Membrane-Bound Polyisoprenoid Quinone under Control of the Proton Motive Force

Next, we evaluated the performance of LAiR in an experimental setting that better reflects the function of an IMP in a physiological context. In living cells, membrane-bound, polyisoprenoid quinones act as the natural substrates of quinone-modifying proteins such as c*bo*_3_, whereas for liposome-reintegrated IMPs typically soluble, non-polyisoprenoid quinones are used as substrates (Figure S4). This can result in substantially altered enzyme catalysis.^26,27^ A second important physiological factor is the proton motive force across the bacterial cytoplasmic membrane, which represents a key effector of respiratory enzymes like c*bo*_3_. Regulation of enzymatic activity by the proton motive force has been described for liposome-reintegrated c*bo*_3_ in a study using a soluble quinone,^28^ but insufficient proton tightness of proteoliposomes precluded investigation in membrane-bound polyisoprenoid quinone systems.^29^ Therefore, we assessed if LAiR allows for incorporation of a membrane-bound quinone and for detection of regulation by the proton motive force.

**Figure 2:**
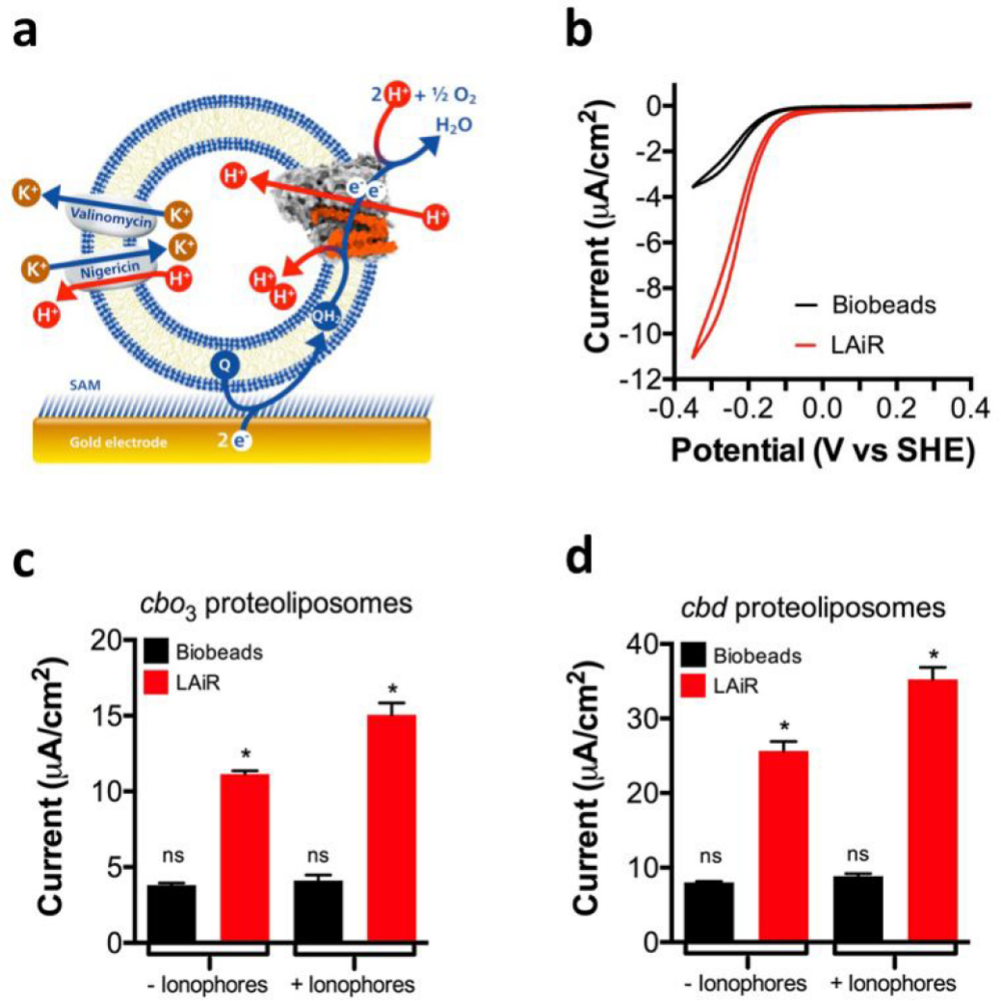
|LAiR visualizes control of membrane-based IMP activity by the proton motive force. a, Scheme showing the experimental setup used to study electron transfer by LAiR-inserted c*bo*_3_ in the presence of a membrane-bound quinone. Proteoliposomes containing c*bo*_3_ are immobilized via a 6-mercaptohexanol self-assembled monolayer (SAM) onto an electrode. The electrode feeds in electrons that reduce a membrane-bound quinone to quinol at the reduction potential of the poly isoprenoid ubiquinone-10 (UQ_10_). The proton motive force generated by c*bo*_3_ can be dissipated by two ionophores, the potassium channel valinomycin together with the H^+^/K^+^ antiporter nigericin. b, Cyclic voltammetry with the immobilized c*bo*_3_ proteoliposomes, showing a comparison of electron transfer activity between LAiR (red curve) and Bio-beads/DDM based reintegration (black curve). c, Impact of the proton motive force on electrochemically detected oxygen consumption activity by cytochrome *bo*_3_ (c*bo*_3_) proteoliposomes. d, Impact of the proton motive force on electrochemically detected oxygen consumption activity by cytochrome *bd* (c*bd)* proteoliposomes. For each experiment 3 biological replicates were used. Shown are either representative results (b) or average values with standard deviation shown (c,d); *p* = <0.021 (c) and <0.028 (d) between LAiR samples ± ionophore addition.

We used LAiR to reintegrate *E. coli* c*bo*_3_ into *E. coli* polar lipid liposomes and immobilized the resulting proteoliposomes on a 6-mercaptohexanol (6MH) modified gold electrode surface via a self-assembled monolayer (SAM, Figure 2a). As a control, we reintegrated *E. coli* c*bo*_3_ using the Bio-beads standard method. To assess the membrane-bound electron transfer activity by c*bo*_3_ we used cyclic voltammetry (Figure 2a). We found that c*bo*_3_ proteoliposomes prepared using LAiR displayed approximately 3-fold higher maximal activity than the control reintegrated using the standard Bio-beads method (Figure 2b,c). Proteoliposomes prepared using LAiR thus display not only enhanced lumen acidification (as shown in Figure 1d,e), but the reintegrated enzyme also retains higher membrane-bound activity as compared to c*bo*_3_ reintegrated using the standard Bio-beads method.

We then evaluated the effect of the proton motive force on the electron transfer activity, a property that has not been measurable to date in the same system.^29^ To dissipate the proton motive force, we added a combination of two ionophores: the K^+^ channel valinomycin and the H^+^/K^+^ antiporter nigericin. The electron transfer activity of the standard Bio-beads/DDM method reintegrated c*bo*_3_ did not significantly change upon addition of valinomycin/nigericin (Figure 2c), indicating that in these proteoliposomes c*bo*_3_*-*catalyzed electron transfer is not noticeably regulated by the proton motive force within the time window measured. In contrast, the electron transfer activity of the c*bo*_3_ proteoliposomes prepared using LAiR considerably increased after the addition of valinomycin/nigericin, demonstrating that the membrane-based electron transfer activity of c*bo*_3_ is under tight control of the proton motive force (Figure 2c).

To exclude that control of membrane-bound activity by the proton motive force represents an idiosyncratic property of c*bo*_3_ proteoliposomes prepared using LAiR, we extended our efforts to cytochrome *bd* (c*bd*) from *E. coli*. c*bd* is a quinol oxidase that reduces oxygen to water and contributes to the proton motive force by vectorial uptake and release of protons, but it is not evolutionary related to c*bo*_3_.^30^ Similar to the results obtained for c*bo*_3_, the membrane-bound electron transfer activity of DDM-purified (c*bd* in solution containing 0.02% DDM), Bio-beads-reintegrated c*bd* displayed only minimal change upon dissipation of the proton motive force within the time window measured, whereas the activity of LAiR-prepared proteoliposomes (from c*bd* in solution containing 0.005% LMNG) considerably increased, revealing tight control by the proton motive force (Figure 2d). Taken together, the results demonstrate that LAiR allows for investigation of IMPs in the context of an ion motive force in membrane platforms utilizing physiological quinones, mimicking the native nonequilibrium biological membrane environment in a living cell.

### Bulk-Phase Evaluation by LAiR Reveals the Regulatory Effects of Electron Supply and Proton-Motive Force on Cytochrome *bo*_3_ Function

We decided to investigate a controversial phenomenon in membrane biochemistry, the so-called proton “leak state” of c*bo*_3_. This proton leak state entails that proton-pumping c*bo*_3_ may conversely also mediate proton backflow across the biomembrane. Previously, using a single-molecule bioelectrochemical platform, Li et al. showed leaking of protons through the membrane mediated by c*bo*_3_. However, this was a rare observation with only 7–8% of c*bo*_3_ molecules actually performing this novel function.^29^ Moreover, this phenomenon had never been observed in the bulk phase. Experimentation at the single molecule level can provide important insights; however, if only a very small subpopulation displays the investigated characteristics, it may be unclear how representative obtained results are. Furthermore, a follow-up study by Berg et al. applied a similar method but did not observe a transmembrane leak-state.^31^ Consequently, it remains an open question whether the *cbo*_3_-mediated proton leak state actually represents a *bona fide* property of this ion-translocating IMP. We reasoned that insufficient proton-tightness of c*bo*_3_ proteoliposomes prepared by standard reintegration methods might be an important factor responsible for the above-mentioned controversial results.

**Figure 3:**
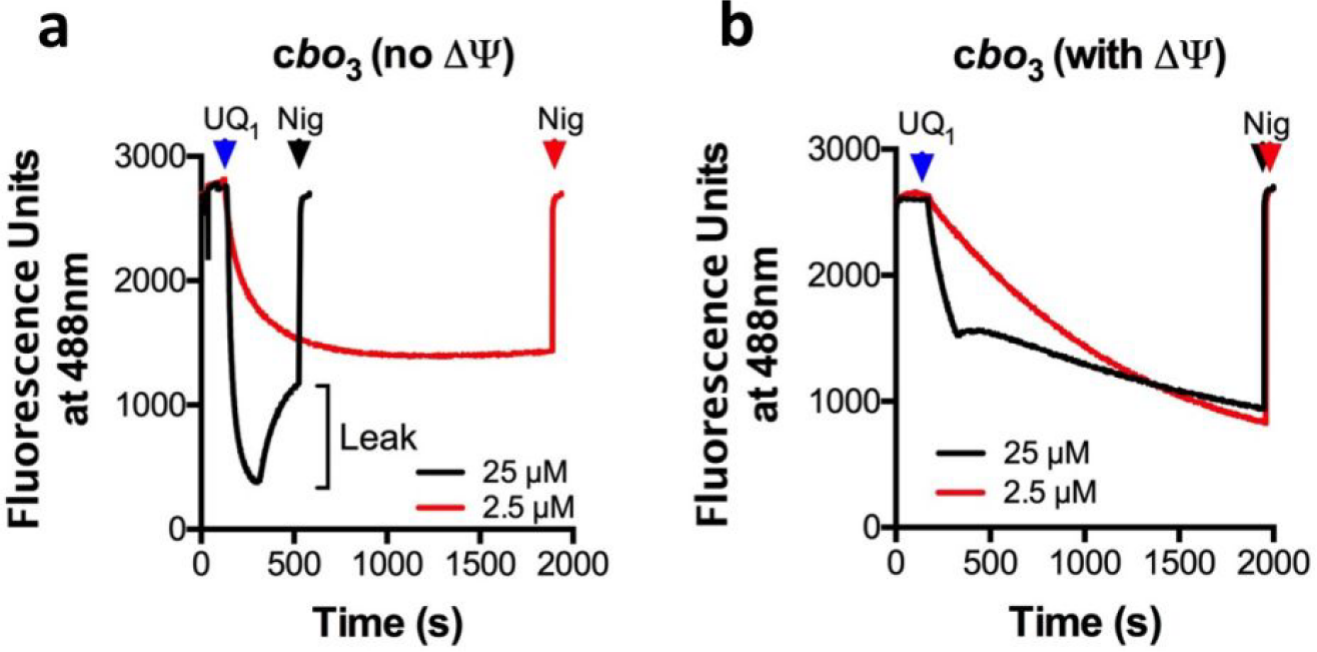
LAiR reveals regulatory mechanisms of cytochrome *bo*_3_. a and b, Liposome acidification by reintegrated c*bo*_3_ monitored by quenching of the ACMA fluorescence using the experimental system shown in Figure 1c. Typically, as protons are pumped into the proteoliposome lumen, an electrochemical gradient is formed across the membrane. Nigericin collapses any H^+^ gradient completely. a, Proton pumping by c*bo*_3_ in the presence of valinomycin. Valinomycin exchanges one K^+^ for every H^+^ entering the proteoliposome, thus abolishes ΔΨ (membrane potential) (i.e., *absence* of back-pressure on *cbo*_3_); b, Proton pumping by c*bo*_3_ in the absence of valinomycin (i.e., *presence* of increasing membrane potential and back-pressure on *cbo*_3_)_._ Various concentrations of Ubiquinol-1 (UQ_1_H_2_) were used to start the reaction as shown. c*bo*_3_ proteoliposomes were prepared using LAiR after a 30 min time period. For each experiment 3 biological replicates were performed with representative results shown.

Due to the high reproducibility and performance of LAiR proteoliposomes, we decided to explore if this new approach enables visualizing the proton leak state at the bulk-phase level, thereby addressing the above-mentioned controversy using LAiR prepared c*bo*_3_ proteoliposomes. Strikingly, at a high quinol concentration a rapid initial proton-pumping rate was followed by a reversal of proton flux out of the vesicle, in line with observations of a proton leak (Figure 3a, black curve). This reversal of quenching had never been observed before in the bulk phase. Further probing of this effect revealed that both the presence and the extent of the c*bo*_3_-mediated proton leak strongly depended on the concentration of the quinol substrate (Figure 3a). We propose that in standard reconstitution systems the large accrual of protons needed to initiate the leak state may not be achieved since proteoliposomes lack sufficient ion tightness (see Figure 1d).

However, in living cells, as ions are accrued across a membrane such as during *cbo*_3_ catalysis, membrane potential also accrues, so we repeated the experiment in the presence of membrane potential to examine physiological relevance (Figure 3b). Under these conditions, a proton leak was not observable at any of the quinol concentrations investigated (Figure 3b), indicating that the physiological relevance of the proton leak state may be limited. However, at high quinol concentration, a sharp deviation in the curve is observed, shifting the proton accumulation in the proteoliposome lumen to a slower rate. Apparently, here the membrane potential drives the transition of *cbo*_3_ into an unknown new state of quinol catalysis.

In summary, LAiR allowed for the investigation of the proton leak state of c*bo*_3_ and its physiological relevance, solving an ongoing dispute in the field. LAiR can enable bulk-phase visualization of complex IMP properties that previously could only be investigated by single-molecule approaches.

### LAiR Allows Nondestructive Reintegration of an IMP into a Pre-Existing Planar Membrane

**Figure 4:**
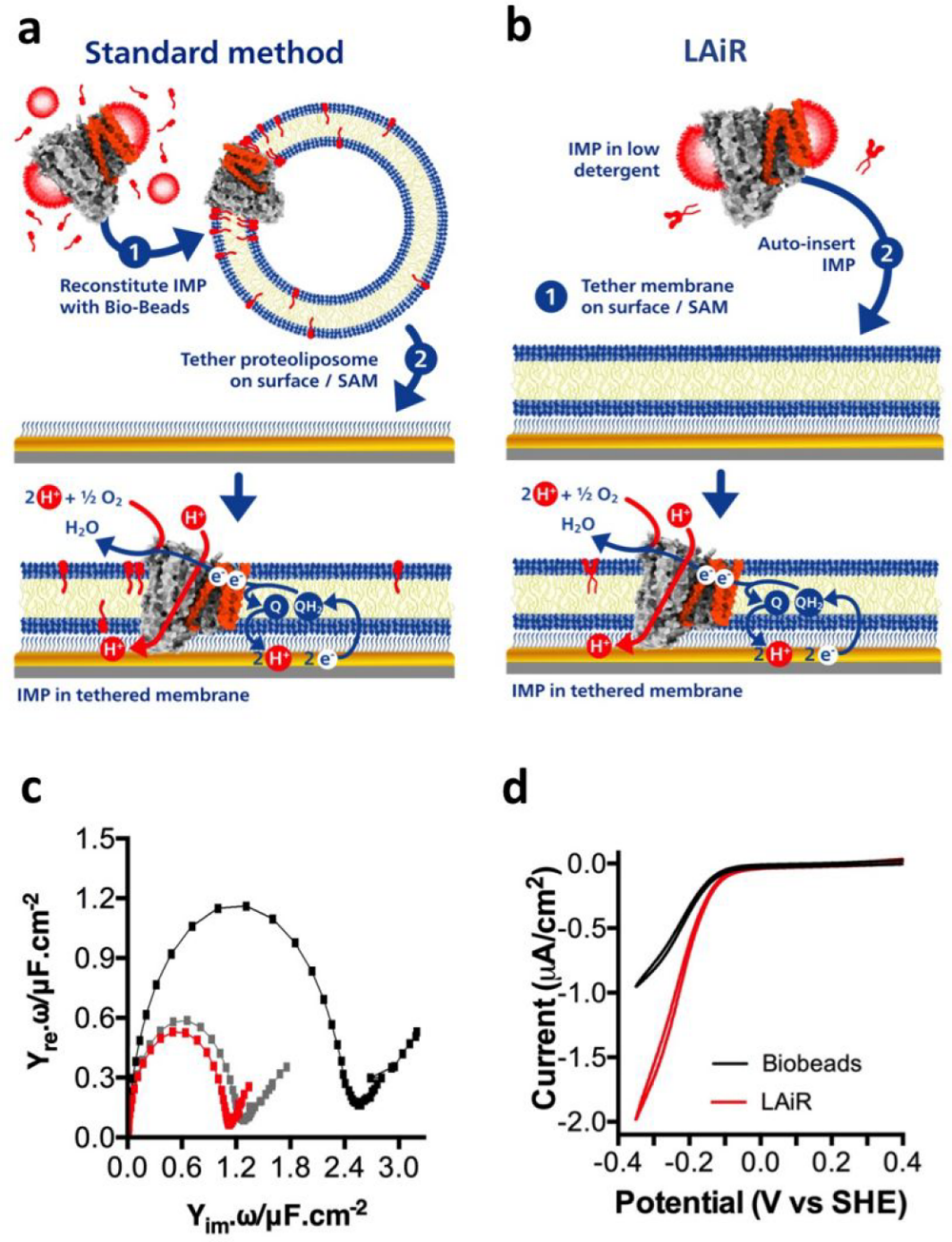
Using LAiR for integration of IMPs into pre-existing planar membranes. a and b, Schemes for integrating an IMP into a surface-tethered membrane (tBLM) using the standard method or LAiR. To assess enzymatic activity, the electrode feeds in electrons that reduce a membrane-bound quinone to quinol at the reduction potential of the poly isoprenoid ubiquinone-10 (UQ_10_; displayed as “Q”). a, Using the standard method, an IMP is first reintegrated into a liposome containing UQ_10_ using either Bio-beads or rapid dilution, and the resulting proteoliposomes are chemically disrupted onto a specialized tethering self-assembled monolayer (SAM). b, Using LAiR, a membrane bilayer is first tethered onto the surface via the self-assembled monolayer in an identical method to a, but without reintegrated protein. Subsequently, the IMP is auto-inserted into the tethered membrane bilayer. c, Impedance spectroscopy of the system shown in b. The black curves show the impedance of the SAM before the membrane bilayer was added, the gray curve shows impedance after membrane bilayer formation, and the red curve is impedance after LAiR auto-insertion of c*bo*_3_. The size of the half-circle is indicative of the accessibility of solution phase ions to the electrode. A large half-circle indicates easy access to the electrode (i.e., no membrane or holes in a membrane) while a small half-circle indicates restricted access (i.e., a tight membrane). d, Cyclic voltammetry comparing between the electrochemically detected oxygen consumption activity of a c*bo*_3_ in a tBLM formed by the Bio-beads/DDM method (black curve) or by LAiR (red curve). For each experiment 3 biological replicates were used; representative results are shown.

Solid-supported lipid membranes and planar bilayers allow for the study of IMPs by analytical tools such as atomic force microscopy, impedance spectroscopy, surface plasmon resonance sensing, and quartz-crystal microbalance.^32^ Currently, to investigate IMPs in these surface-tethered bilayers, the proteins are first reintegrated into liposomes, and in a second step the resulting proteoliposomes are disrupted to form a bilayer containing a membrane-bound quinone on a gold electrode surface modified with a tethering self-assembled monolayer (SAM) (Figure 4a).^33^ In order to further evaluate the scope of LAiR, we explored its applicability to pre-existing membrane bilayers tethered onto an electrode surface (Figure 4b). To demonstrate this utility, we assembled a tethered membrane bilayer using liposomes composed of *E. coli* polar lipids containing substrate and then used LAiR to reintegrate c*bo*_3_ (c*bo*_3_ in solution in LMNG; see “LAiR” in Methods for specific conditions) (Figure 4b). As a control, we reintegrated c*bo*_3_ into liposomes of identical composition using a standard Bio-beads/DDM protocol and then assembled a tethered membrane bilayer using these proteoliposomes (Figure 4a).

To evaluate membrane protein reintegration into the tethered membrane by LAiR, we assessed the permeability of the tethered membrane using impedance spectroscopy. Formation of the tethered membrane bilayer decreased the permeability as revealed by a drop in capacitance (Figure 4c). Interestingly, upon reintegration of c*bo*_3_ using LAiR, the capacitance marginally decreased further (Figure 4c), suggesting that protein integration into the preformed membrane was not only nondestructive, but the insertion of IMP with bound LMNG appeared to tighten the membrane. Cyclic voltammetry revealed that the activity of *cbo*_3_ reintegrated using LAiR into the preformed tethered membrane was 2-fold higher than that observed with a membrane formed using a membrane assembled from c*bo*_3_ proteoliposomes prepared by Bio-beads reintegration (Figure 4d), confirming highly effective c*bo*_3_ insertion. This demonstrates that LAiR not only can integrate protein into a preformed tethered membrane in a rapid, nondestructive manner, but also the protein’s activity with a physiologically relevant quinone substrate is significantly higher than that in current state-of-the-art experimental set-ups. As such, LAiR can serve as a versatile method to integrate IMPs into biomimetic drug screening platforms and biosensors.

### LAiR Preserves Intactness and Higher-Order Oligomeric Features of a Fragile Multisubunit IMP

**Figure 5:**
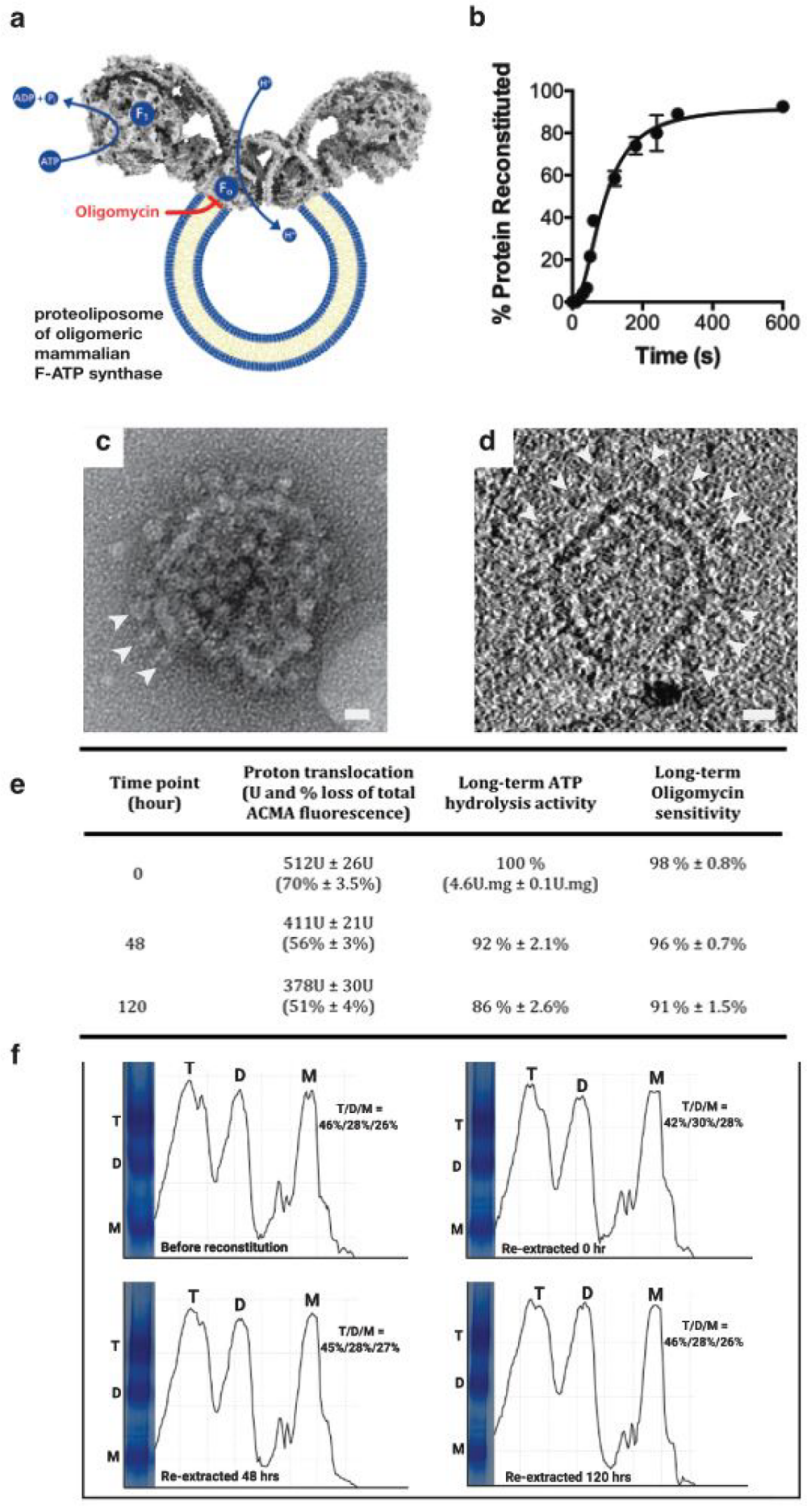
Structural integrity of a fragile multisubunit IMP is maintained by LAiR. a, Scheme of a mammalian F-type ATP synthase dimer and the reactions it catalyzes. The F_1_ part, where ATP hydrolysis occurs, and the transmembrane F_0_ domain, where the inhibitor oligomycin binds, are indicated. b, Time-course of reintegration. F-type ATP synthase purified from bovine heart mitochondria was reintegrated into liposomes composed of bovine heart lipids. c and d, Reintegration of F-type ATP synthase complexes into the proteoliposomes as assessed by negative-stain (c) or cryo electron tomography after a 30 min LAiR treatment. c, Negative-stained intact proteoliposome, and d, tomographic slice of a proteoliposome (also see SI Movie 1). White arrows indicate bovine F-type ATP synthase complexes decorating the proteoliposomes in both c and d. e, Long-term proteoliposome lumen acidification and connectivity between the F_1_ and the F_0_ domain of the reintegrated F-type ATP synthase. The proteoliposomes with reintegrated ATP synthase were stored on ice at 4 °C over a period of 120 h (5 days). Proton pumping by the ATP synthase and proteoliposome stability was evaluated by measuring acidification of the liposome lumen upon hydrolysis of ATP using the fluorescent dye ACMA (for a representative time course, please see Figure S5). Quenching of ACMA fluorescence is presented in both total units (U) of fluorescence quenched at 488 nm and the percentage of quenching at different time points. ATP hydrolysis was measured coupled to NADH oxidation (via the pyruvate kinase and lactate dehydrogenase reactions), whose absorption at 340 nm was monitored (for a representative time course, please see Figure S5). f, Long-term stability of reintegrated F-type ATP synthase dimers and tetramers. After LAiR, the F-type ATP synthase was re-extracted from the proteoliposomes at the indicated time points and the relative abundance of tetramers (T), dimers (D), and monomers (M) was assessed by Native PAGE. For each experiment, at least 2 biological replicates were used. Average values with standard deviation (b, e) or representative results (c,d,f) are shown.

To evaluate if LAiR can also be utilized for highly complex multisubunit IMPs of mammalian origin, we assessed the impact of LAiR on the intactness of F-type ATP synthase from bovine heart mitochondria. The mammalian F-type ATP synthase is a large (MW > 600 kDa) fragile IMP complex comprising 28 subunits in its monomeric form, displaying a characteristic shape amenable to observation of intactness by electron microscopy. It consists of two distinct domains, F_1_, which catalyzes ATP hydrolysis (or synthesis, depending on the direction of the reaction), and the membrane bound F_0_ domain_,_ which, coupled to ATP hydrolysis, conducts protons across the membrane (Figure 5a).^34^ In mammalian mitochondria, the F-type ATP synthase forms oligomeric supercomplexes of rows of dimers which are located at the ridge of cristae and bend the membrane by ∼85°, i.e., representing a crucial molecular player in cristae architecture.^35,36^

We purified F-type ATP synthase from bovine heart mitochondria^37^ and used LAiR to reintegrate the sample (bovine F-type ATP synthase in solution containing 0.005% LMNG) into liposomes prepared from bovine heart lipids. Similar to c*bo*_3_, preparation of F-type ATP synthase proteoliposomes using LAiR was virtually completed within several minutes (Figure 5b). Negative stain electron microscopy (EM) and cryo electron tomography (cryoET) revealed the F-type ATP synthase complexes tightly packed in the liposomes (Figure 5c,d, SI Movies 1 and 2). Clearly, LAiR is not restricted to prokaryotic systems but is also applicable to IMPs and lipids extracted from eukaryotic sources.

A key problem in the investigation of F-type ATP synthase is that upon exposure to detergents, during liposome reintegration or upon long-term storage, the enzyme is prone to a functional disconnection between its two domains, thereby uncoupling proton flow in the F_0_ domain from ATP hydrolysis in the F_1_ domain.^38^ In this regard, EM is a highly suitable tool for detecting even miniscule amounts of broken F-ATP synthase complexes. Strikingly, the LAiR-inserted F-type ATP synthase displayed the F_1_ domains decorating the proteoliposomes at high density, in the absence of naked F_0_ domains (Figure 5c,d). Moreover, the F_0_ and F_1_ domains maintained strong functional connectivity, even after long-term incubation on ice for 5 days, as revealed by the pronounced liposome lumen acidification and almost complete inhibition by the F_0_ inhibitor oligomycin A, corroborating the absence of dissociated F_1_ domains (Figure 5a, 5e, and Figure S5). These results demonstrate that even a fragile multisubunit IMP can be stably reintegrated into liposomes using LAiR and remain intact over extended periods.

A second key feature of mitochondrial F-type ATP synthase, itself a multisubunit complex, is its ability to form higher-order assemblies such as dimers or tetramers in the inner mitochondrial membrane.^35^ Higher-order structural features can strongly influence the functionality of an IMP. In the case of mitochondrial F-type ATP synthase formation of dimers is instrumental for folding the cristae structure of the inner membrane,^35,36^ a key factor in mitochondrial physiological function. However, mitochondrial F-type ATP synthase is prone to dissociation into monomers in the presence of DDM,^39^ and proper liposome reconstitution of purified dimeric F-type ATP synthase is challenging.^40,41^ We confirmed that LAiR reintegration of bovine heart F-type ATP synthase did not cause noticeable dissociation of dimers or tetramers into monomers (Figure 5f). Native PAGE demonstrated that the monomer/dimer/tetramer ratio of the auto-inserted enzyme was maintained over a period of 5 days when stored on ice at 4 °C (Figure 5f). As such, LAiR enables long-term investigation of complex mammalian IMPs without loss of their higher-order oligomeric state.

## Discussion

Our results demonstrate that LAiR reintegration of IMPs is highly rapid and allows for detection and study of IMP properties that are not accessible with standard methods. LAiR results in proteoliposomes having remarkable reproducibility and stability over time and maintains even fragile IMPs in an active, stable state. The functional intactness exhibited by proteoliposomes comprising IMPs and lipids from prokaryotic or eukaryotic sources opens hitherto closed doors to the investigation of a broad scope of problems in biomembrane research. The long-term stability associated with LAiR enables the researcher to perform work at ease, spreading experimentation on an individual sample over several days with intermediate storage on ice. As the approach is technically straightforward, LAiR can likely be adopted in various laboratories without extensive method-specific training and is expected to be scalable for industrial use.

The phenomenon of auto-insertion of integral membrane proteins into preformed lipid bilayers has been known for more than 50 years and its mechanism ascribed to the presence of destabilizing membrane defects that resemble those necessary for lipid bilayer fusion (for a comprehensive review see ref (42)). The necessity for membrane defects induced by conventional detergents, reflected in the lack of functional data in previous reports^42^ and the low proton tightness we observed for proteoliposomes prepared with DDM or digitonin, is likely the main reason that auto-insertion, while known for several decades, never gained much traction in the field of biomembrane research.

The molecular processes during LAiR and the factors responsible for the observed proteoliposome lumen acidification and high enzymatic activity are not well understood yet. High liposome lumen acidification may be attributable to a variety of factors, including (i) better activity and/or stability of the enzyme, (ii) higher reconstitution efficiency, (iii) a more uniform orientation of the membrane in the liposomes, (iv) a more uniform reconstitution of the IMPs across all liposomes, or (v) a lower proton permeability. In this regard, the observed pronounced long-term stability of LAiR-proteoliposomes suggests a highly intact liposome membrane with only little proton backflow. In addition, the decreased capacitance detected in planar bilayers indicates a tightening of the membrane upon LAiR reintegration, which we ascribe to the doping of the membrane by carry-over LMNG from the IMP bound detergent micelle which dissolves into the membrane after auto-insertion. However, it certainly cannot be excluded that the alternative factors specified above significantly contribute to the high degree of acidification observed for proteoliposomes prepared by LAiR.

Overall it is plausible that the almost complete absence of free detergent micelles in solutions of IMPs stabilized by less than 0.005% LMNG together with the lipid-like molecular architecture of LMNG are key factors that enable the extraordinary tightness and stability of LAiR proteoliposomes.^20,43^ Moreover, as revealed by the low performance of proteoliposomes prepared with GDN, the specific combination of polar headgroup and hydrophobic tail of the detergent molecule likely plays an important role in proteoliposome formation, e.g., for bridging the IMP with the lipid bilayer. During random collision events such a bridging facilitated by LMNG may enable the temporary destabilization of the lipid bilayer necessary for IMP insertion. Post insertion, the lipid like architecture of LMNG might then allow for optimal bilayer stability and proton tightness. A systematic comparison of different detergents or detergent-derivatives (e.g., using derivatives that have been reported for LMNG^19^ or can be synthesized in the future) might shed more light on the process of membrane auto-insertion and on factors underlying the orientation of the reintegrated IMPs, thereby further improving the performance of this new method.

We have demonstrated that LAiR is an effective method capable of exploring molecular mechanisms of IMP function that are not accessible with standard approaches. We characterized the membrane-bound activity of two quinone-utilizing enzymes and show that this activity is under control of the proton motive force. We also demonstrated that the proposed c*bo*_3_ transmembrane proton leak state can exist and is regulated by the quinol substrate, but was not observable under physiological membrane potential conditions, resolving an open question in the field. We expect that LAiR can be utilized to study the role of ion leak in a variety of IMPs, e.g., in transporters or channels. We also demonstrate that LAiR enabled the bulk-phase investigation of biochemical phenomena that hitherto could only be addressed by specialized single molecule approaches. In this regard, LAiR may open up new avenues for the discovery of currently unknown functions critical to IMP biology.

LAiR will broaden the utility of membrane biochemistry and may also prove useful in fields such as electrochemistry, electrophysiology, and cryo-EM of proteoliposomes. Next to the importance for fundamental research, the expected scalability of the LAiR approach and the high stability of proteoliposomes comprising LAiR reintegrated multisubunit IMPs can also enable long-term usage in applied settings, e.g., in biosensor systems based on surface-tethered membranes, for drug delivery or in liposome-based vaccine formulations. Finally, LAiR also has a strong potential to provide input for efforts in synthetic biology aimed at constructing artificial, liposome-based systems mimicking cell-like functions,^44^ and might even allow for integration of IMPs into native cell membranes to modify cell function.

## Materials and Methods

### Chemicals

β-Dodecyl-maltoside (DDM, Anagrade, <0.2% α-configuration), glyco-diosgenin (GDN), and lauryl maltose neopentyl glycol (LMNG) were purchased from Anatrace. Lipids were purchased from Avanti Polar Lipids. Other chemicals were obtained from Sigma.

### Purification of Cytochrome *bo*_3_ from *Escherichia coli*

Cytochrome *bo*_3_ (c*bo*_3_) was extracted and purified from inner membranes based on Asseri et al.^45^^45^*E. coli* was aerobically grown to mid log phase at 37 °C in Luria–Bertani (LB) medium supplemented with 500 μM CuSO_4_ and 100 μg mL^–1^ carbenicillin. Cells were harvested by centrifugation at 10,000 × *g* for 10 min. The pellet was then washed and repelleted twice with buffer B (20 mM 3-*N*-morpholino-propanesulfonic acid (MOPS), 30 mM Na_2_SO_4_, 10% Glycerol pH 7.4). Cells were then resuspended in buffer B containing 1 Roche complete mini protease inhibitor tablet per 50 mL, 0.1 mM phenylmethylsulfonyl fluoride, 0.1 mg pancreatic DNase per mL, and lysed by two passages through a French press at 20,000 psi. Any remaining debris and unbroken cells were removed by centrifugation at 10,000 × *g* for 30 min. The supernatant was then ultracentrifuged (200,000 × *g*, 45 min, 4 °C) and the membrane pellet resuspended in buffer C (20 mM MOPS, 30 mM Na_2_SO_4_, 25% w/w sucrose, pH 7.4). This was applied to the top of a 30% w/w to 55% w/w sucrose gradient and ultracentrifugation (130,000 × *g*, 16 h, 4 °C) with no deceleration or breaking to separate inner membrane from outer membrane. The inner membrane fraction was removed from the sucrose gradient and washed 3 times with buffer B by ultracentrifugation (200,000 × *g*, 45 min, 4 °C). Inner membranes were then resuspended in buffer B and either used immediately for purification or stored in aliquots at −80 °C until use. To extract c*bo*_3_, inner membrane vesicles were diluted to 5 mg of protein mL in solubilization buffer (20 mM Tris HCl, pH 8.0, 5 mM MgSO_4_, 10% glycerol, 0.5% LMNG/GDN/Digitonin or 1% DDM, 300 mM NaCl, 10 mM imidazole) and incubated at 30 °C for 30 min with gentle inversion every 5 min. The unsolubilized material was removed by ultracentrifugation (180,000 × *g*, 45 min, 4 °C), and the supernatant was applied to a Nickel-Sepharose High Performance (GE Healthcare) column that was previously washed with water and equilibrated with IMAC buffer (50 mM Tris HCl, pH 8.0, 5 mM MgSO_4_, 10% glycerol, 0.005% LMNG or GDN, or 0.05% DDM or 0.02% Digitonin, 300 mM NaCl) containing 10 mM imidazole. To remove contaminating proteins, the resin was washed with IMAC buffer containing 30 mM imidazole and 150 mM NaCl, and c*bo*_3_ was eluted with IMAC buffer containing 200 mM imidazole, 150 mM NaCl, and 20% glycerol, 0.005% LMNG or GDN, or 0.05% DDM or 0.02% Digitonin. The red c*bo*_3_ containing fractions were pooled and concentrated using an Amicon Ultra centrifugal filter device (molecular weight cutoff (MWCO), 100,000). Fractions containing c*bo*_3_ (10 mg/mL) aliquots of ∼30 μL were flash frozen in liquid nitrogen and stored at −80 °C until further use.

### Buffer Exchange of *cbo*_3_ Purified in LMNG for Digitonin

*cbo*_3_ in 200 mM imidazole, 150 mM NaCl, and 20% glycerol, 0.005% LMNG was exchanged in a PD Minitrap G-25 (GE Healthcare) column according to manufacturers instructions to 200 mM imidazole, 150 mM NaCl, and 20% glycerol, 0.02% Digitonin.

### Purification of cytochrome *bd* from *Escherichia coli*

Cytochrome *bd* (c*bd*) was purified from *E. coli* based on Goojani et al.^46^ Briefly, *E. coli* strain MB43 carrying the pET17cydABX-Strep-tag plasmid was grown in Luria–Bertani medium with 100 μg/mL Ampicillin at 37 °C overnight with shaking at 200 rpm. The bacteria were diluted to OD_600_ 0.01 in 800 mL LB medium with 100 μg/mL Ampicillin and incubated until reaching OD_600_ 0.4. Then IPTG (0.45 mM final conc.) was added and the bacteria were incubated again at 37 °C, 200 rpm until reaching OD_600_ 2.0. Cells were sedimented by centrifugation at 6,000 × *g* for 20 min (JA-10 rotor). The pellets were washed by phosphate buffer saline, pH 7.4, and pelleted at 6,000 × *g* for 20 min. Approximately 15 g of wet cells were resuspended with 75 mL of MOPS solution (50 mM MOPS, 100 mM NaCl and protease inhibitor (cOmplete EDTA free)). The cells were disrupted by three passages though a Stansted cell homogenizer at 18,000 psi. Unbroken cells were centrifuged at 9,500 × *g* for 20 min. Subsequently, the supernatant was pelleted by ultracentrifugation 250,000 × *g* for 75 min at 4 °C. The pellet was resuspended in MOPS solution and the protein concentration was determined. To extract c*bd*, the protein concentration was adjusted to 10 mg/mL with MOPS solution and either DDM (1% final conc.) or LMNG (0.5% final conc.) were added and the solution was incubated at 4 °C for an hour with gentle shaking. Unsolubilized material was sedimented by ultracentrifugation at 250,000 × *g* at 4 °C for 15 min (70-Ti rotor). The collected supernatant was applied on a Strep-Tactin XT column (IBA Lifesciences) at 4 °C. To remove nonspecifically bound protein, the column was washed with 50 mM sodium phosphate pH 8.0 containing 300 mM NaCl, protease inhibitor (cOmplete EDTA free), and 0.01% DDM or 0.005% LMNG. Finally, the protein was eluted from the column with 50 mM sodium phosphate pH 8.0 containing 300 mM NaCl, protease inhibitor (cOmplete EDTA free), 0.02% DDM or 0.005% LMNG, and 2.5 mM desthiobiotin. The c*bd* containing fractions were pooled and concentrated using an Amicon Ultra centrifugal filter device (molecular weight cutoff (MWCO), 100,000). Fractions containing c*bd* (10 mg/mL) aliquots of ∼30 μL were flash frozen in liquid nitrogen and stored at −80 °C until further use.

### Purification of F-type ATP Synthase from *Bos taurus* (Bovine)

Purification of mammalian F-type ATP synthase was conducted as previously described.^20,36^ Fresh *B. taurus* hearts were obtained immediately after slaughter by an authorized slaughterhouse and inner mitochondrial membranes were purified according to Shimada et al.^47^ Fat and connective tissues were carefully removed allowing the preparation of 1,000 g of minced meat. Each 500 g was suspended in 3,250 mL of 23 mM sodium phosphate buffer (pH 7.4 at 0 °C) and homogenized for 5 min at 13,000 rpm in a homogenizer (Nihon Seiki), followed by centrifugation for 20 min at 2,800 rpm in a refrigerated centrifuge (Kubota Model 9810; RS-6600 rotor). The precipitate was suspended in 3,375 mL of 22.2 mM sodium phosphate buffer (pH 7.4) and rehomogenized, followed by centrifugation for 20 min at 2,800 rpm as previously. Supernatants were then combined and centrifuged for a further 30 min at 10,000 rpm with a refrigerated centrifuge (Beckman Model Avanti HP-30I) using a JLA-10.500 rotor. The precipitate was then suspended in 50 mM Tris-HCl buffer (pH 8.0) and pelleted for 30 min at 30,000 rpm with an ultracentrifuge (Beckman Model-7) using a 45 Ti rotor. The pellet was suspended in 50 mM Tris-HCl buffer (pH 8.0) containing 660 mM sucrose to a final protein concentration of ∼23 mg/mL. The suspension was kept in a 40 mM HEPES buffer (pH 7.8) containing 2 mM MgCl_2_, 0.1 mM EDTA, and 0.1 mM DTT and solubilized on ice via slow addition of deoxycholate and decylmaltoside to final concentrations of 0.7% (wt/vol) and 0.4% (wt/vol), respectively. The suspension was then centrifuged at 176,000 × *g* for 50 min and the supernatant applied to a sucrose step gradient (40 mM HEPES pH 7.8, 0.1 mM EDTA, 0.1 mM DTT, 0.2 wt %/vol decylmaltoside and 2.0, 1.1, 1.0, or 0.9 M sucrose) and centrifuged at 176,000 × *g* for 15.5 h. Fractions exhibiting ATP hydrolysis activity were loaded onto a Poros-20HQ ion-exchange column. The detergent was exchanged to LMNG using a double gradient from 0.2% to 0% decyl-maltoside and 0%–0.05% LMNG for 80 min at 1 mL/min. Complexes were eluted by a linear concentration gradient of 0–240 mM KCl in 40 mM HEPES pH 7.8, 150 mM sucrose, 2 mM MgCl_2_, 0.1 mM EDTA, 0.1 mM DTT, and 0.005% (wt/vol) LMNG. Fractions containing bovine F-type ATP synthase (10 mg/mL) aliquots of less than 500 μL were flash frozen in liquid nitrogen and stored at −80 °C until further use.

### Protein Quantification

Protein concentrations were determined using a bicinchoninic acid (BCA) protein assay kit (Sigma) with bovine serum albumin as the standard.

For assessment of the effectiveness of reintegration of IMPs two methods were used concurrently. To measure protein content in proteoliposomes, first reconstitution solutions containing proteoliposomes were pelleted by centrifugation for 10 min on a benchtop centrifuge at 13,000 rpm. The supernatant protein content was then assessed using a BCA assay and the pelleted proteoliposomes resuspended and transferred to a 0.2 μm filter for assessment of protein content using a Schaffner-Weissmann assay.^48^

### SDS Polyacrylamide Gel Electrophoresis (PAGE) and Blue-Native PAGE

For all purified proteins used in this study the purification quality was routinely assessed by SDS polyacrylamide gel electrophoresis (SDS-PAGE). For *E. coli* c*bo*_3_ in digitonin after buffer exchange, the quality after buffer exchange was routinely assessed by polyacrylamide gel electrophoresis next to the LMNG sample from which it was derived. Results from representative gels are shown in Figure S6 (*E. coli* c*bo*_3_), Figure S7 (*E. coli* c*bd*), and Figure S8 (bovine F-ATP synthase).

For Blue-Native PAGE after LAiR, F-type ATP synthase was extracted from the proteoliposomes at the indicated time points using 0.5% LMNG. After centrifugation at 20,000 × *g* for 10 min at 4 °C, the supernatants were prepared for and separated by 3–12% Bis-Tris Mini-gels (Novex Life Technologies) according to the manufacturer’s instructions. ATP synthase bands were analyzed using ImageStudioLite software.

### Lipid Treatment and Proteoliposome Preparation

Lipids used in this study were purchased from Avanti Polar Lipids, Inc., Alabaster, AL. Lipids were stored and treated as in McMillan et al.^49^ Stock solutions of either native *E. coli* polar lipids extract (ECPL) or bovine heart lipids (BHPL) were dried under an N_2_ stream. Where appropriate, ubiquinone (UQ_10_) was added to the chloroform-dissolved lipid mixtures at 1% mass/mass and dried together. Liposomes were resuspended in 20 mM Tris(hydroxymethyl)aminomethane hydrochloride (Tris-HCl, Sigma) buffer containing 100 mM KCl. All liposome concentrations after resuspension were 10 mg/mL. These were then extruded 11 times through a 400 nm track-etched membrane using an extruder (Avanti Polar Lipids, Inc., Alabaster, AL).

### Reintegration of IMPs into Liposomes

#### Bio-beads Method

Lipids (8 mg/mL) in reintegration buffer (20 mM MOPS (pH 7.4), 30 mM Na_2_SO_4_, 100 mM KCl) containing 55 mM octyl glucoside were briefly ultrasonicated. Purified IMP (in 0.05% DDM) was added to a final protein concentration of 0.2 mg/mL (final conc. DDM = 0.0015% DDM), and the mixture was slowly stirred for 15 min at 20 °C. Activated SM^2^ Bio-beads (80 mg wet beads/mL) were added directly to the mixture, and stirring continued for 30 min. After this, another 80 mg/mL Bio-beads were added followed by another 30 min incubation. Finally, another 160 mg/mL Bio-beads were added followed by a 90 min incubation to attempt to complete the removal of detergent. The top phase with the proteoliposomes was carefully removed with a pipet tip that did not pass any Bio-beads and diluted 10-fold with reintegration buffer and ultracentrifuged at 180,000 × *g* for 60 min. The pellet was resuspended in reintegration buffer and used as indicated in figures.

#### Rapid Dilution Method

Lipids (8 mg/mL) in reintegration buffer (20 mM MOPS (pH 7.4), 30 mM Na_2_SO_4_, 100 mM KCl) containing 55 mM octyl glucoside were briefly ultrasonicated. Purified IMP (in 0.05% DDM) was added to a final concentration of 0.2 mg/mL protein (final conc. DDM = 0.0015% DDM), and the mixture was slowly stirred for 60 min at 20 °C. The mixture was then diluted 200-fold in reintegration buffer and ultracentrifuged at 180,000 × *g* for 60 min. The pellet was resuspended in reintegration buffer and used as indicated in figures.

#### LAiR (LMNG Auto-insertion Reintegration)

Lipids (10 mg/mL) in reintegration buffer (20 mM MOPS (pH 7.4) 30 mM Na_2_SO_4_, 100 mM KCl) were extruded 13 times through a 400 nm polycarbonate membrane. This liposome solution was then mixed with the purified IMP (solubilized in in 0.005% LMNG). The final concentration of protein was 0.2 mg/mL (5 μL protein used), and 9.75 mg/mL lipids (200 μL used), resulting in a final concentration of 0.00015% LMNG. The mixture was slowly inverted for 5–30 min (as indicated in figures) at 20 °C. The reconstitution was then either used immediately or diluted 10-fold and ultracentrifuged at 180,000 × *g* for 30 min (as indicated). If the latter treatment was used, the pellet was resuspended in reintegration buffer and used as indicated in figures. Where LAiR was used in a planar bilayer system, after formation of the planar bilayer, 2 μL of protein was applied 5 mm above the bilayer in solution and allowed to incubate at 20 °C for 20 min. After this, the complete electrochemical cell was washed out and assays performed.

#### Autoinsertion Method Using DDM, GDN, or Digitonin

Lipids (10 mg/mL) in reintegration buffer (20 mM MOPS (pH 7.4) 30 mM Na_2_SO_4_, 100 mM KCl) were extruded 13 times through a 400 nm polycarbonate membrane. This liposome solution was then mixed with the purified IMP (solubilized in either 0.05% DDM, 0.005% GDN, or 0.02% digitonin). The final concentration of protein was 0.2 mg/mL (5 μL protein solution was used, independent of the detergent), and 9.75 mg/mL lipids (200 μL used), resulting in a final concentration of 0.0015% DDM, or 0.00015% GDN, or 0.0007% digitonin. The mixtures were then slowly inverted for 5–30 min (as indicated in figures) at 20 °C. The reconstitution was then either used immediately or diluted 10-fold and ultracentrifuged at 180,000 × *g* for 30 min (as indicated). If the latter treatment was used, the pellet was resuspended in reintegration buffer and used as indicated in figures.

### Biochemical Assays

#### ATP Hydrolysis Activity after Reconstitution

ATP hydrolysis activity was measured at 38 °C with stirring at 1,000 rpm using an ATP regenerating assay.^50^ The assay mixture contained 50 mM MOPS (pH 7.4), 30 mM NaCl, 100 mM KCl, 3 mM phosphoenolpyruvate, 1.5 mM MgCl_2_, 0.25 mM NADH, 1 μM valinomycin, 0.57 U/mL pyruvate kinase, 3.2 U/mL lactate dehydrogenase, and 2 mM ATP. The reaction was initiated by the addition of 10 μg of auto-inserted ATP synthase into 1 mL of assay mixture. The rate of NADH oxidation was monitored continuously at 340 nm using a modified Cary 60 spectrophotometer (Agilent). Where indicated 2 μM oligomycin was added. The activity that hydrolyzed 1 μmol of ATP per min is defined as 1 unit. The activity of reconstituted ATP synthase at day 0 was 4.6 U/mg protein.

#### ATP-Dependent Liposome Acidification (ACMA Fluorescence Quenching)

ATP-dependent proton translocation was determined at 38 °C based on the quenching of ACMA. The 1.5-mL reaction mixture contained 50 mM MOPS (pH 7.4), 30 mM NaCl, 100 mM KCl, 3 mM phospho*enol*pyruvate, 1.5 mM MgCl_2_ 0.25 mM NADH, 0.57 U/mL pyruvate kinase, 3.2 U/mL lactate dehydrogenase, 1 μM ACMA, 1 μM valinomycin, and 10 μg F_1_F_0_ ATP synthase complexes reintegrated in bovine heart lipid liposomes. After the fluorescence signal had stabilized, the reaction was initiated by the addition of 2.5 mM neutralized ATP. Fluorescence was measured with an excitation wavelength of 410 nm and an emission wavelength of 480 nm (slit width, 10 nm) in a modified Cary Eclipse photospectrophotometer (Agilent). ATP-dependent quenching of ACMA fluorescence at day 0 was 75%.

#### Quinone-Dependent Liposome Acidification (ACMA Fluorescence Quenching)

Cytochrome *bo*_3_ (c*bo*_3_) proton translocation was tracked in a method similar to that in Hards et al.^51^ Briefly, proteoliposomes (0.2 mg) consisting of 2% c*bo*_3_/mass *E. coli* polar lipids doped with 1% mass ubiquinone-10 (UQ_10_) per mL were prewarmed to 37 °C for 15 min in 20 mM MOPS pH 7.4, 30 mM Na_2_SO_4_, 100 mM KCl, 1 mM DTT, and 1 μM ACMA ± 1 μM valinomycin with vigorous stirring (800 rpm). In Figure 3, 2 μM ACMA was used and valinomycin was not used when examining the effect of membrane potential. Quenching was initiated by the addition of 2.5 μM ubiquinone-1 (UQ_1_) in ethanol (or as indicated) and reversed using 0.75 μM of the ionophore nigericin as indicated in text. Ethanol controls had no effect on ACMA quenching.

### Electrochemical Assays

Electrochemical experiments in tethered bilayer lipid membranes and proteoliposomes were prepared as described elsewhere.^52^ All the experiments were carried out with ultraflat template stripped gold (TSG) working electrodes. 150 nm of 99.99% gold (Goodfellows) was evaporated on silicon wafers using a Telemescal evaporator at <2 × 10^–6^ mbar. 1.2 cm^2^ glass slides were glued to the gold layer with Epo-Tek 377 and cured for 2 h at 120 °C. The TSG surface was exposed by detaching the glass slides from the silicon wafers before use. The formation of the self-assembled monolayers (SAMs) containing the cholesterol “tether” and the formation of the SSM onto the electrode were performed as described previously.^33^ SAMs were formed by incubating a freshly exposed TSG slide in 0.11 mM EO3-cholesteryl and 0.89 mM 6-mercaptohexanol (6MH) in propanol for 16 h. Where vesicles/proteoliposomes were used as described previously,^53^ SAMs were made with 1 mM 6MH alone. After incubation, the excess thiol was gently washed away with isopropanol and methanol, and the electrodes were then dried in a stream of N_2_. For bilayer SAMs this procedure results in an approximate 60%/40% EO3-cholesteryl/6-mercaptohexanol area ratio on the surface as confirmed by impedance spectroscopy before each experiment. For SAMs prepared for vesicle/proteoliposome experiments, the surface was coated in 100% 6MH. To form tethered lipid membranes (tBLMs), vesicles or proteoliposomes were added to the SAM surface at a final concentration of 0.5 mg/mL in the presence of 10 mM CaCl_2_ and incubated for 1 h until a capacitance drop to less than 1.2 μF/cm^2^ was observed. The surface was then rinsed three times with water, then buffer containing 0.5 mM EDTA to remove any traces of calcium ions in the cell. In vesicle/proteoliposome experiments, either vesicles or proteoliposomes were added to the 100% 6MH SAM surface at a final concentration of 0.5 mg/mL and incubated for 30 min. Finally, the SAM-modified or vesicle/proteoliposome-modified electrodes were rinsed three times with buffer and used in the electrochemistry experiments. Care was taken to keep the electrodes immersed in an aqueous environment at all times during rinsing. The time window for measurable proton leak is 25 s from quinone reduction on the forward scan to the start of the reverse scan (i.e., the point at which the electrode starts to reduce membrane-bound ubiquinone so that cytochromes can use the reduced quinone to reduce oxygen).

### Quantification of Detergents by LC-MS/MS

Protein extracts and detergent standards were analyzed using a UPLC BEH (1.0 × 100 mm, 1.7 μm, Waters, Acquity) separation phase coupled online to an ESI-Q-TOF mass spectrometer (Q-TOF Premier, Waters Micromass, Manchester, UK) operated in ESI+ mode, alternating full scan and selected reaction monitoring (SRM) modes. After sample injection, a constant flow at 50 μL/min of a solvent consisting of 15% B was kept over 3 min, followed by a linear gradient to a solvent composition of 95% B over 16 min. Finally, the flow was kept constant at 95% B over additional 4 min until the back-equilibration to solvent start conditions. The flow rate of 50 μL/min was maintained using an Acquity Ultra Performance LC pump system (Waters, Milford, United States). Buffer A consisted of 50 mM ammonium acetate in LC-MS grade water and buffer B consisted of 5% mobile phase A in LC-MS grade acetonitrile. During all analysis runs, samples were cooled at 10 °C and the analytical separation column was maintained at 25 °C. The full scan was acquired over the mass range of 150–1250 *m*/*z* at 1 s scan times, and the selected reaction monitoring (SRM) modes isolated mass windows at 528.3 *m*/*z*, for dodecyl-d-maltoside (DDM) and 1005.6 *m*/*z* for lauryl maltose neopentyl glycol (LMNG), over 1 s scan time each. A collision energy (CID) of 9 eV was used for selected reaction monitoring. The Q-TOF mass spectrometer was calibrated using Glu1-Fibrinopeptide B (human, Sigma-Aldrich) before analysis. External calibration for detergents was performing by injecting a dilution series of every detergent (DDM: 3.82^–3^ to 5.0^–5^ μmol on column injection, LMNG: 1.6^–4^ to 4.0^–5^ μmol on column injection) to the LC-ESI-Q-TOF analysis system. All standards and samples were analyzed in duplicates and summed peak area counts of the major fragment of the respective selected reaction monitoring trace were used to establish a calibration curve and to further determine the concentration of the detergents in the protein extracts. Data analysis was performed manually using MassLynx 4.1, and determination of calibration curves and calculation of detergent concentrations in samples was performed using Microsoft Excel (for calibration curves see Supporting Information material).

### Negative Stain EM of Liposome-Reintegrated *B. taurus* F-type ATP Synthase

A 2.5 μL aliquot was applied onto freshly glow-discharged, carbon-coated 400 mesh copper grids (Veco). After brief blotting (Whatman #1), the samples were stained by using a 2% uranyl acetate solution and air-dried. Images were taken with a JEM1010 transmission electron microscope (JEOL) equipped with a 4 × 4 K Tietz CMOS TemCamF416 (TVIPS, Gauting, Germany) at 100 kV.

### Cryo-Electron Tomography of LAiR Reintegrated *B. taurus* F-type ATP Synthase

QUANTIFOIL (R0.6/1, Mo) grids with gold colloidal markers on the carbon films were used for cryo-EM. The grids were coated with poly(l-lysine) (BBI Solutions) for 10 min after 1 min glow-discharging and then 15 nm colloidal golds (Sigma-Aldrich) were applied for 10 min. The solution was removed from the grids with filter paper and then the grids were washed by distilled water. The grids were coated with a 10- to 20-nm-thick layer of carbon after drying. 3 μL of sample was applied onto the grid and blotted for 6 s with a blot force of 10 at 4 °C and 100% humidity using a Vitrobot (Thermo Fisher) and then flash-frozen by plunging into liquid ethane. The samples were observed with a Titan Krios (Thermo Fisher) operating at 300 kV and equipped with a Falcon II detector at a direct magnification of 22.5K. The images were taken from 0° to +70° and then from −2° to −70° with 2° steps. The defocus values were about 5 μm and the electron dose for each exposure was 1.6 e^–^/Å^2^ with a total dose of less than 120 e^–^/Å^2^. Tilt series were aligned using the gold fiducials and back-projected to generate tomographic volumes using the IMOD package^54^ with a pixel size of 5.7 Å.

All studies reported here studies have complied with all relevant ethical regulations for animal testing and research.

## Data Availability

All data are available in the main text or the Supporting Information.

## Supporting Information Available

Reintegration efficiency of current standard methods; DDM auto-insertion reintegration; Detergent structures; Structures of soluble vs membrane bound ubiquinone; ACMA quenching and ATP hydrolysis by F-ATP synthase in proteoliposomes produced by LAiR at time point zero hours; SDS-PAGE of cytochrome *bo3* from *E. coli* purified with various detergents; SDS-PAGE of F-type ATP synthase from *Bos taurus* purified with LMNG (PDF)
Movie 1: Cryo electron tomogram z-slices of bovine F-ATP synthase proteoliposome cryo electron tomogram shown in Figure 5d (MP4)
Movie 2: Cryo electron tomogram z-slices of several bovine F-ATP synthase proteoliposome of the same cryo-grid used for the production of Figure 5d (MP4)

## References

[ref1] Santos R.; et al. A comprehensive map of molecular drug targets. Nat. Rev. Drug Discov 2017, 16, 19–34. 10.1038/nrd.2016.230. 27910877 PMC6314433

[ref2] Renaud J. P.; et al. Cryo-EM in drug discovery: achievements, limitations and prospects. Nat. Rev. Drug Discov 2018, 17, 471–492. 10.1038/nrd.2018.77. 29880918

[ref3] Palsdottir H.; Hunte C. Lipids in membrane protein structures. Biochim. Biophys. Acta 2004, 1666, 2–18. 10.1016/j.bbamem.2004.06.012. 15519305

[ref4] Popot J. L. Amphipols, nanodiscs, and fluorinated surfactants: three nonconventional approaches to studying membrane proteins in aqueous solutions. Annu. Rev. Biochem. 2010, 79, 737–775. 10.1146/annurev.biochem.052208.114057. 20307193

[ref5] McLean M. A.; Gregory M. C.; Sligar S. G. Nanodiscs: A Controlled Bilayer Surface for the Study of Membrane Proteins. Annu. Rev. Biophys 2018, 47, 107–124. 10.1146/annurev-biophys-070816-033620. 29494254 PMC6370528

[ref6] Amati A. M.; Graf S.; Deutschmann S.; Dolder N.; von Ballmoos C. Current problems and future avenues in proteoliposome research. Biochem. Soc. Trans. 2020, 48 (4), 1473–92. 10.1042/BST20190966. 32830854

[ref7] Rigaud J. L.; Pitard B.; Levy D. Reconstitution of membrane proteins into liposomes: application to energy-transducing membrane proteins. Biochim. Biophys. Acta 1995, 1231, 223–246. 10.1016/0005-2728(95)00091-V. 7578213

[ref8] Skrzypek R.; Iqbal S.; Callaghan R. Methods of reconstitution to investigate membrane protein function. Methods 2018, 147, 126–141. 10.1016/j.ymeth.2018.02.012. 29454861

[ref9] Althoff T.; Davies K. M.; Schulze S.; Joos F.; Kuhlbrandt W. GRecon: a method for the lipid reconstitution of membrane proteins. Angew. Chem., Int. Ed. Engl. 2012, 51, 8343–8347. 10.1002/anie.201202094. 22821803 PMC3494379

[ref10] Schaedler T. A.; Tong Z.; van Veen H. W. The multidrug transporter LmrP protein mediates selective calcium efflux. J. Biol. Chem. 2012, 287, 27682–27690. 10.1074/jbc.M112.372334. 22730320 PMC3431714

[ref11] Bald D.; et al. ATP synthesis by F_0_F_1_-ATP synthase independent of noncatalytic nucleotide binding sites and insensitive to azide inhibition. J. Biol. Chem. 1998, 273, 865–870. 10.1074/jbc.273.2.865. 9422743

[ref12] Zhou X.; Graham T. R. Reconstitution of phospholipid translocase activity with purified Drs2p, a type-IV P-type ATPase from budding yeast. Proc. Natl. Acad. Sci. U. S. A. 2009, 106, 16586–16591. 10.1073/pnas.0904293106. 19805341 PMC2757829

[ref13] Hankamer B.; Morris E.; Nield J.; Gerle C.; Barber J. Three-dimensional structure of the photosystem II core dimer of higher plants determined by electron microscopy. J. Struct Biol. 2001, 135, 262–269. 10.1006/jsbi.2001.4405. 11722166

[ref14] Niroomand H.; Mukherjee D.; Khomami B. Tuning the photoexcitation response of cyanobacterial Photosystem I via reconstitution into Proteoliposomes. Sci. Rep 2017, 7, 2492 10.1038/s41598-017-02746-5. 28559589 PMC5449388

[ref15] Crouch C. H.; et al. Optimization of Detergent-Mediated Reconstitution of Influenza A M2 Protein into Proteoliposomes. Membranes (Basel) 2018, 8, 103 10.3390/membranes8040103. 30413063 PMC6315538

[ref16] Stetsenko A.; Guskov A. An Overview of the Top Ten Detergents Used for Membrane Protein Crystallization. Crystals 2017, 7, 197 10.3390/cryst7070197.

[ref17] Arias-Cartin R.; Grimaldi S.; Arnoux P.; Guigliarelli B.; Magalon A. Cardiolipin binding in bacterial respiratory complexes: structural and functional implications. Biochim. Biophys. Acta 2012, 1817, 1937–1949. 10.1016/j.bbabio.2012.04.005. 22561115

[ref18] Yankovskaya V.; et al. Architecture of succinate dehydrogenase and reactive oxygen species generation. Science 2003, 299, 700–704. 10.1126/science.1079605. 12560550

[ref19] Chae P. S.; et al. Maltose–neopentyl glycol (MNG) amphiphiles for solubilization, stabilization and crystallization of membrane proteins. Nat. Methods 2010, 7, 1003–1008. 10.1038/nmeth.1526. 21037590 PMC3063152

[ref20] Hauer F.; et al. GraDeR: Membrane Protein Complex Preparation for Single-Particle Cryo-EM. Structure 2015, 23, 1769–1775. 10.1016/j.str.2015.06.029. 26278176

[ref21] Chung K. Y.; et al. Role of detergents in conformational exchange of a G protein-coupled receptor. J. Biol. Chem. 2012, 287, 36305–36311. 10.1074/jbc.M112.406371. 22893704 PMC3476297

[ref22] Schägger H. Respiratory chain supercomplexes of mitochondria and bacteria. Biochim et Biophys Acta-Bioenergetics. 2002, 1555, 154–159. 10.1016/S0005-2728(02)00271-2. 12206908

[ref23] VanAken T.; Foxall-VanAken S.; Castleman S.; Ferguson-Miller S. Methods Enzymol. 1986, 125, 27–35. 10.1016/S0076-6879(86)25005-3. 3012259

[ref24] Le Bon C.; Michon B.; Popot J. L.; Zoonens M. Amphipathic environments for determining the structure of membrane proteins by single-particle electron cryo-microscopy. Q. Rev. Biophys. 2021, 54, e6 10.1017/S0033583521000044. 33785082

[ref25] Chae P. S.; Rasmussen S. G.; Rana R. R.; Gotfryd K.; Kruse A. C.; Manglik A.; Cho K. H.; Nurva S.; Gether U.; Guan L.; Loland C. J.; Byrne B.; Kobilka B. K.; Gellman S. H. A new class of amphiphiles bearing rigid hydrophobic groups for solubilization and stabilization of membrane proteins. Chemistry. 2012, 18, 9485–9490. 10.1002/chem.201200069. 22730191 PMC3493560

[ref26] Godoy-Hernandez A.; McMillan D. G. G. The profound influence of lipid composition on the catalysis of the peripheral membrane NADH Type-II Oxidoreductase. Membranes 2021, 11 (5), 363 10.3390/membranes11050363. 34067848 PMC8156991

[ref27] Futami A.; Hurt E.; Hauska G. Vectorial redox reactions of physiological quinones. Requirement of a minimum length of the isoprenoid side chain. Biochim. Biophys. Acta 1979, 547, 583–596. 10.1016/0005-2728(79)90035-5. 486435

[ref28] Verkhovskaya M. L.; Garcia-Horsman A.; Puustinen A.; Rigaud J.-L.; Morgan J. E.; Verkhovsky M. I.; Wikstrom M. Glutamic acid 286 in subunit I of cytochrome *bo*_3_ is involved in proton translocation. Proc. Natl. Acad. Sci. U. S. A. 1997, 94, 10128 10.1073/pnas.94.19.10128. 9294174 PMC23326

[ref29] Li M.; et al. Single Enzyme Experiments Reveal a Long-Lifetime Proton Leak State in a Heme-Copper Oxidase. J. Am. Chem. Soc. 2015, 137, 16055–16063. 10.1021/jacs.5b08798. 26618221 PMC4697922

[ref30] Safarian S.; et al. Structure of a *bd* oxidase indicates similar mechanisms for membrane-integrated oxygen reductases. Science 2016, 352, 583–586. 10.1126/science.aaf2477. 27126043 PMC5515584

[ref31] Berg J.; Block S.; Höök F.; Brzezinski P. Single Proteoliposomes with *E. coli* Quinol Oxidase: Proton Pumping without Transmembrane Leaks. Isr J. Chem. 2017, 57, 437–445. 10.1002/ijch.201600138.

[ref32] Knoll W.; et al. Functional tethered lipid bilayers. J. Biotechnol. 2000, 74, 137–158. 10.1016/S1389-0352(00)00012-X. 11143794

[ref33] McMillan D. G. G.; et al. Protein-protein interaction regulates the direction of catalysis and electron transfer in a redox enzyme complex. J. Am. Chem. Soc. 2013, 135, 10550–10556. 10.1021/ja405072z. 23799249 PMC3823026

[ref34] Noji H.; Ueno H.; McMillan D. G. G. Catalytic robustness and torque generation of the F1-ATPase. Biophys Rev. 2017, 9, 103–118. 10.1007/s12551-017-0262-x. 28424741 PMC5380711

[ref35] Davies K. M.; et al. Macromolecular organization of ATP synthase and complex I in whole mitochondria. Proc. Natl. Acad. Sci. U. S. A. 2011, 108, 14121–14126. 10.1073/pnas.1103621108. 21836051 PMC3161574

[ref36] Jiko C.; et al. Bovine F_1_F_0_ ATP synthase monomers bend the lipid bilayer in 2D membrane crystals. Elife 2015, 4, e06119 10.7554/eLife.06119. 25815585 PMC4413875

[ref37] Urbani A.; et al. Purified F-ATP synthase forms a Ca(2+)-dependent high-conductance channel matching the mitochondrial permeability transition pore. Nat. Commun. 2019, 10, 4341 10.1038/s41467-019-12331-1. 31554800 PMC6761146

[ref38] Runswick M. J.; et al. The affinity purification and characterization of ATP synthase complexes from mitochondria. Open Biol. 2013, 3, 120160 10.1098/rsob.120160. 23407638 PMC3603449

[ref39] Meyer B.; et al. Identification of two proteins associated with mammalian ATP synthase. Mol. & Cell Proteomics. 2007, 6, 1690–1699. 10.1074/mcp.M700097-MCP200. 17575325

[ref40] Mnatsakanyan N.; et al. A mitochondrial megachannel resides in monomeric F1FO ATP synthase. Nat. Commun. 2019, 10, 5823 10.1038/s41467-019-13766-2. 31862883 PMC6925261

[ref41] Blum T. B.; Hahn A.; Meier T.; Davies K. M.; Kuhlbrandt W. Dimers of mitochondrial ATP synthase induce membrane curvature and self-assemble into rows. Proc. Natl. Acad. Sci. U S A 2019, 116, 4250–4255. 10.1073/pnas.1816556116. 30760595 PMC6410833

[ref42] Jain M. K.; Zakim D. The spontaneous incorporation of proteins into preformed bilayers. Biochim. Biophys. Acta 1987, 906, 33–68. 10.1016/0304-4157(87)90004-9. 3032257

[ref43] Singh S. K.; Sigworth F. J. Cryo-EM: Spinning the Micelles Away. Structure 2015, 23, 1561 10.1016/j.str.2015.08.001. 26331455 PMC4901521

[ref44] Berhanu S.; Ueda T.; Kuruma Y. Artificial photosynthetic cell producing energy for protein synthesis. Nat. Commun. 2019, 10, 1325 10.1038/s41467-019-09147-4. 30902985 PMC6430821

[ref45] Asseri A. H.; et al. Cardiolipin enhances the enzymatic activity of cytochrome *bd* and cytochrome *bo*_3_ solubilized in dodecyl-maltoside. Sci. Rep 2021, 11, 8006 10.1038/s41598-021-87354-0. 33850195 PMC8044227

[ref46] Goojani H. G.; et al. The carboxy-terminal insert in the Q-loop is needed for functionality of *Escherichia coli* cytochrome *bd*-I. Biochim Biophys Acta Bioenerg 2020, 1861, 148175 10.1016/j.bbabio.2020.148175. 32061652

[ref47] Shimada S.; et al. Solubilization conditions for bovine heart mitochondrial membranes allow selective purification of large quantities of respiratory complexes I, III, and V. Protein Expr Purif 2018, 150, 33–43. 10.1016/j.pep.2018.04.015. 29702187

[ref48] Schaffner W.; Weissmann C. A rapid, sensitive, and specific method for determination of protein in dilute solutions. Anal. Biochem. 1973, 56, 502–514. 10.1016/0003-2697(73)90217-0. 4128882

[ref49] McMillan D. G. G.; Marritt S. J.; Butt J. N.; Jeuken L. J. Menaquinone-7 is specific cofactor in tetraheme quinol dehydrogenase CymA. J. Biol. Chem. 2012, 287, 14215–14225. 10.1074/jbc.M112.348813. 22393052 PMC3340143

[ref50] McMillan D. G. G.; Keis S.; Dimroth P.; Cook G. M. A specific adaptation in the a subunit of thermoalkaliphilic F_1_F_0_-ATP synthase enables ATP synthesis at high pH but not at neutral pH values. J. Biol. Chem. 2007, 282, 17395–17404. 10.1074/jbc.M611709200. 17434874

[ref51] Hards K.; et al. Ionophoric effects of the antitubercular drug bedaquiline. Proc. Natl. Acad. Sci. U. S. A. 2018, 115, 7326–7331. 10.1073/pnas.1803723115. 29941569 PMC6048524

[ref52] Godoy-Hernandez A.; Tate D. J.; McMillan D. G. G. Revealing the Membrane-Bound Catalytic Oxidation of NADH by the Drug Target Type-II NADH Dehydrogenase. Biochemistry 2019, 58, 4272–4275. 10.1021/acs.biochem.9b00752. 31592658 PMC6812066

[ref53] McMillan D. G.G.; Marritt S. J.; Kemp G. L.; Gordon-Brown P.; Butt J. N.; Jeuken L. J.C. The impact of enzyme orientation and electrode topology on the catalytic activity of adsorbed redox enzymes. Electrochim. Acta 2013, 110, 79–85. 10.1016/j.electacta.2013.01.153. 24634538 PMC3951721

[ref54] Kremer J. R.; Mastronarde D. N.; McIntosh J. R. Computer visualization of three-dimensional image data using IMOD. J. Struct Biol. 1996, 116, 71–76. 10.1006/jsbi.1996.0013. 8742726

