# Supplementary material for: Rapid and Highly Stable Membrane Reconstitution by LAiR Enables the Study of Physiological Integral Membrane Protein Functions
Source: ACS Cent Sci. 2023 Feb 22;9(3):494–507. doi: 10.1021/acscentsci.2c01170 (PMC10037447; doi:10.1021/acscentsci.2c01170)
Supplement: Supplementary file 1 — oc2c01170_si_001.pdf [file oc2c01170_si_001.pdf]

Supplementary Materials for

Rapid and highly stable membrane reconstitution by LAiR enables the study of physiological integral membrane protein functions

Albert Godoy-Hernandez, Amer H. Asseri, Aiden J. Purugganan, Chimari Jiko, Carol de Ram, Holger Lill, Martin Pabst, Kaoru Mitsuoka, Christoph Gerle*, Dirk Bald* & Duncan G. G. McMillan*

*Corresponding authors. Email: d.g.g.mcmillan@tudelft.nl, d.bald@vu.nl, or christoph.gerle@riken.jp

This PDF file includes:

Figs. S1 to S8

Other Supplementary Materials for this manuscript include the following:

Movies S1 to S2

Fig. S1.

Reintegration efficiency of current standard methods.

Reintegration efficiency (100% represents the total protein amount present in the sample) for DDM-purified c*bo*$_3$ into *E. coli* polar lipids using either biobeads (>1.5 hrs) or rapid dilution (>1 hr). Quantification was initially ascertained using a Schaffner-Weissmann assay in membranes and solution, to confirm the utility of a BCA assay for only protein left in solution to increase the rapid nature of the compete LAiR process and this control. For each experiment 3 biological replicates were used, shown are either average values with standard deviation.

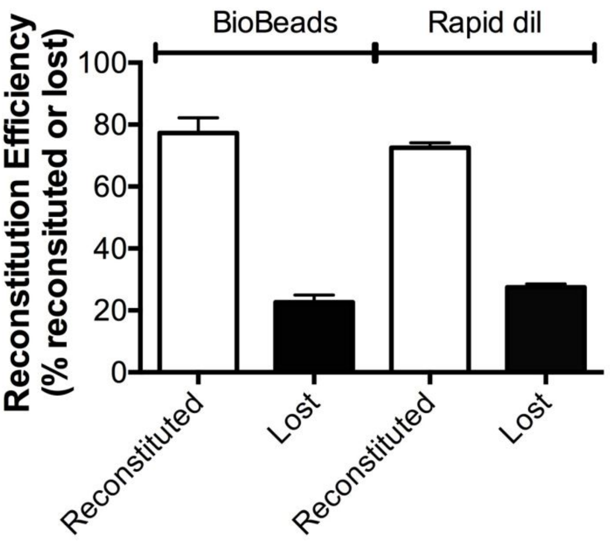

Fig. S2.

DDM Auto-insertion Reintegration. **a,** Schematic view of the standard reintegration method, where biobeads are used to reduce detergent concentration, in comparison with the DDM Auto-insertion method. **b,** Time course of reintegrating c*bo*$_3$ purified using LMNG into *E. coli* polar lipids liposomes using auto-insertion. For each experiment 3 biological replicates were used, shown are either average values with standard deviation.

a

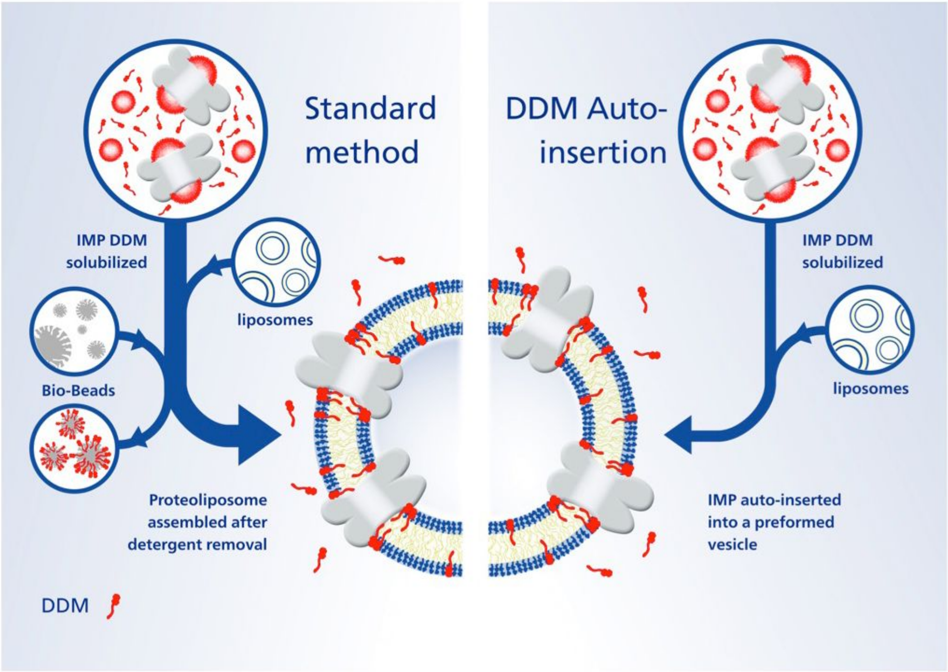

b

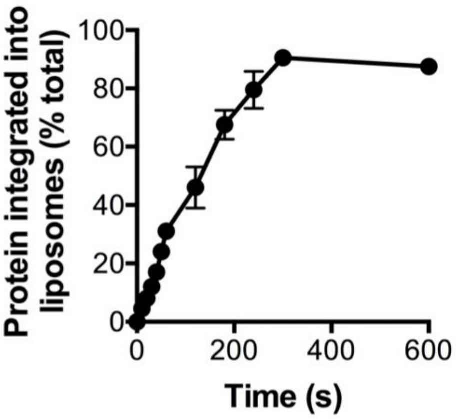

Fig. S3.

Detergent structures. Structures of five different detergents that are especially useful to membrane protein biology and examined in this study on their potential for auto-insertion mediated reconstitution of integral membrane proteins into preformed liposomes. **a,** Digitonin; **b,** Glyco-diosgenin (GDN); **c,** lauryl maltose neopentyl glycol (LMNG); **d,** β-dodecyl-maltoside (DDM) and e, n-octyl-β-D-glucopyranoside (OGP)

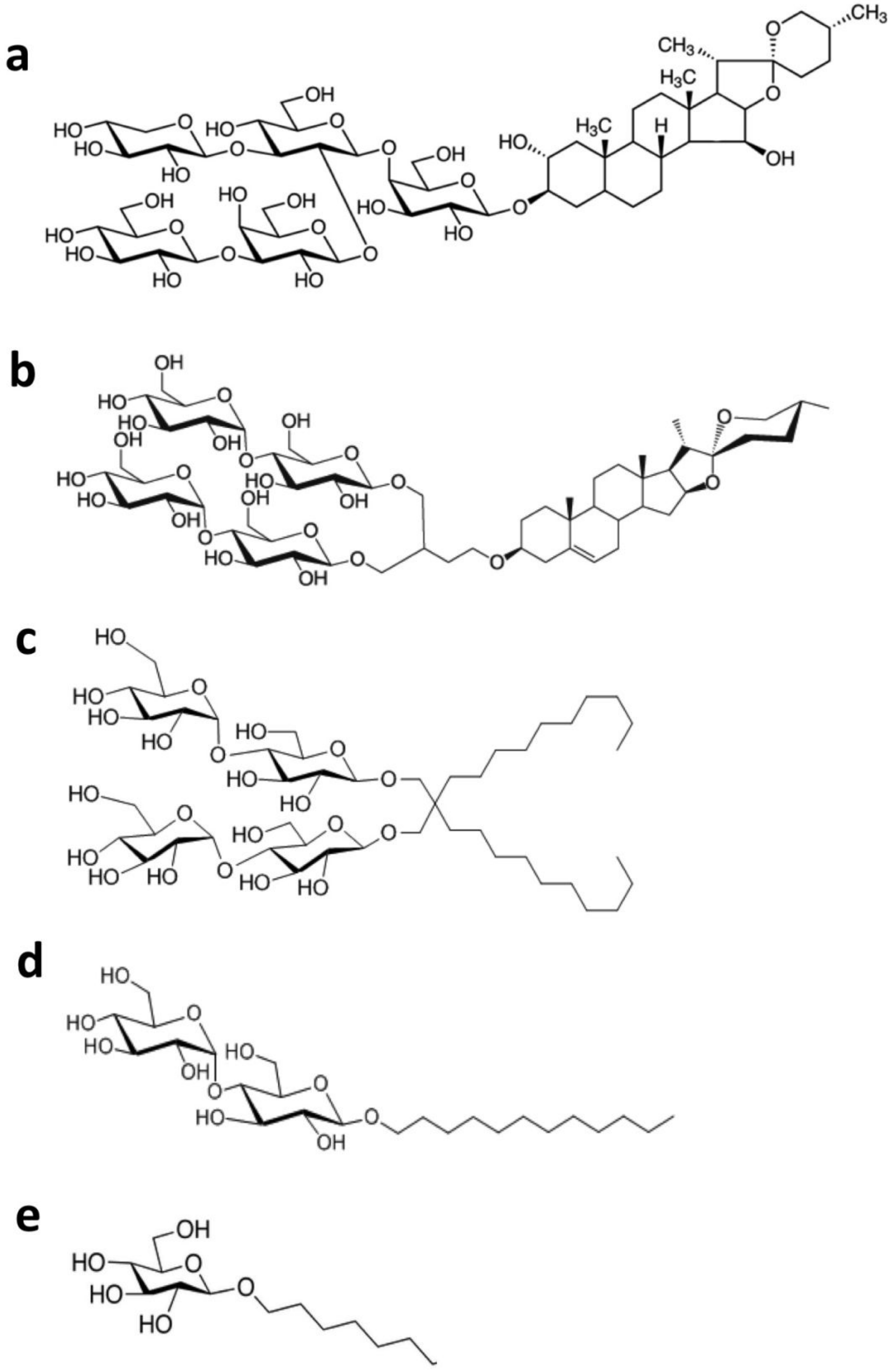

Fig. S4. Structures of soluble *vs* membrane bound ubiquinone.

a, Soluble ubiquinone (no isoprenoid tail); **b,** membrane-bound quinone (poly-isoprenoid tail; typically 7-9 units in biological systems)

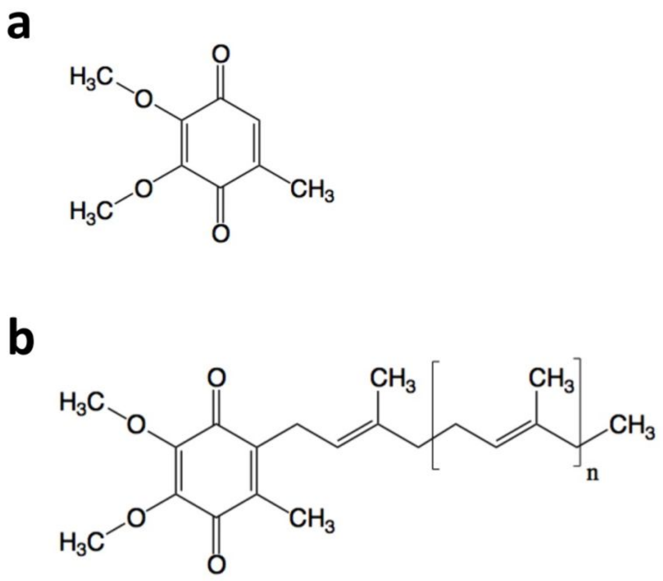

Fig. S5.

ACMA quenching and ATP hydrolysis by F-ATP synthase in proteoliposomes produced by LAiR at time point zero hours.

a, Representative trace of ACMA-quenching assay involving bovine F_1F_o ATP synthase reintegrated into bovine heart lipids using LAiR at time point zero hours. Protons pumped into the proteoliposome lumen is driven by ATP hydrolysis causes an acidification, driving ACMA to accumulated inside the proteoliposome thus quenching the fluorescence signal. Maximum quench is where the proton pump rate into the proteoliposome lumen is equal to the proton leak rate out of the proteoliposome lumen. The reaction was initiated by the addition of 2.5 mM ATP and the proton gradient established abolished by the addition of 0.75 µM nigericin. **b,** Representative trace of an NADH oxidation-coupled enzymatic assay involving bovine F_1F_o ATP synthase reintegrated into bovine heart lipids using LAiR at time point zero hours. When ATP is consumed it is linked to the loss of NADH absorbance at 340 nm. Each trace is representative of three experimental replicates.

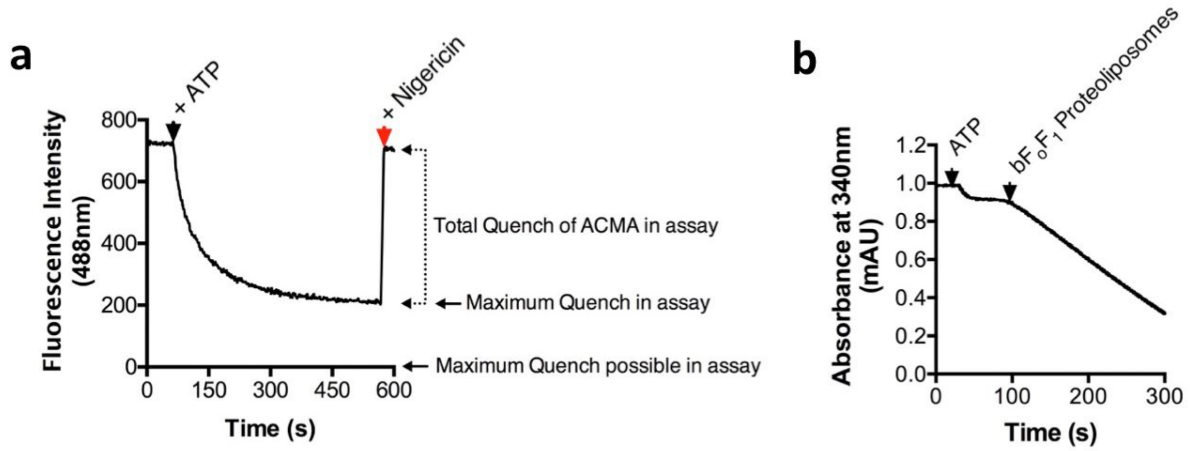

Fig. S6.

SDS-PAGE of cytochrome *bo₃* from *E. coli* purified with various detergents

Mini-Protean TGX gels (4-12%, BIO-RAD) were used, protein bands were visualized by coomassie staining. Left lane: Marker (Precision Plus protein markers, BIO-RAD), lane 1: cytochrome bo_3 purified with GDN, lane 2: cytochrome bo_3 purified with LMNG, lane 3: cytochrome bo_3 purified with DDM, lane 4: cytochrome bo_3 purified with LMNG and buffer exchanged to Digitonin, lane 5: Fully functional cytochrome bo_3 purified with LMNG that was used for buffer exchange to Digitonin. Subunits I-IV represent subunits of cytochrome bo_3.

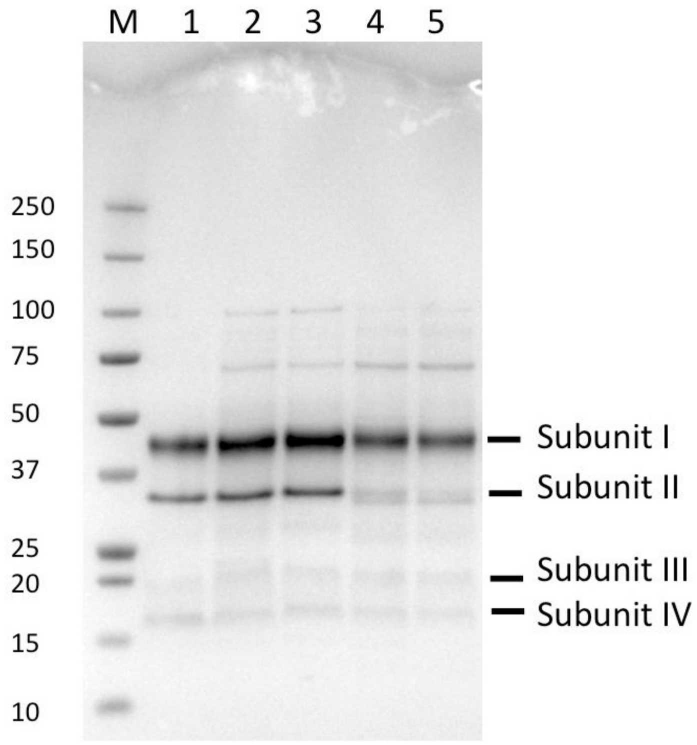

Fig. S7.

SDS-PAGE of cytochrome *bd* from *E. coli* purified with either LMNG or DDM.

Mini-Protean TGX gels (4-20%, BIO-RAD) were used, protein bands were visualized by silver staining. Left lane: cytochrome *bd* purified with LMNG, middle lane: marker (Rainbow marker, full range, Merck), right lane: cytochrome *bd* purified with DDM. CydA and CydB represent subunits of cytochrome *bd*.

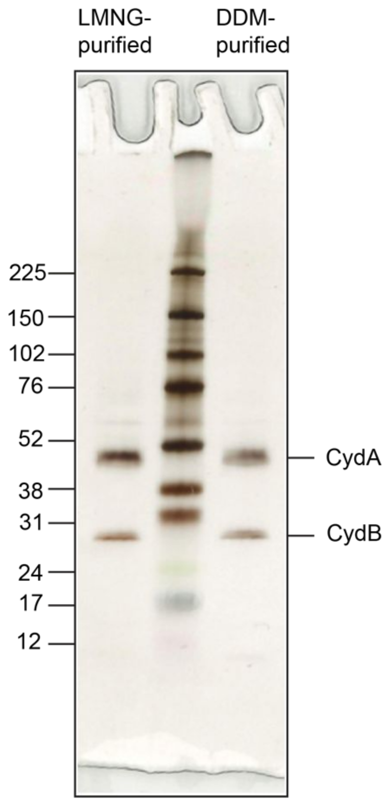

Fig. S8.
SDS-PAGE of F-type ATP synthase from *Bos taurus* purified with LMNG.
e-PAGEL gels (10-20%, ATTO) were used, protein bands were visualized by SimplyBlue SafeStain (Invitrogen). Left lane: molecular weight marker (EzProtein Ladder, ATTO), right lane: F-type ATP synthase from *Bos taurus* purified with LMNG.

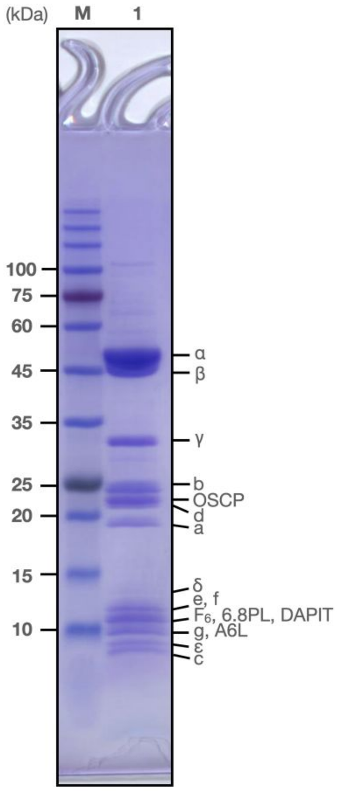

Movie S1.
Cryo electron tomogram z-slices of bovine F-ATP synthase proteoliposome cryo electron tomogram shown in Figure 5d.

Movie S2.
Cryo electron tomogram z-slices of several bovine F-ATP synthase proteoliposome of the same cryo-grid used for the production of Figure 5d.
